# Characterization of Endoglucanase (GH9) Gene Family in Tomato and Its Expression in Response to *Rhizophagus irregularis* and *Sclerotinia sclerotiorum*

**DOI:** 10.3390/plants14223458

**Published:** 2025-11-12

**Authors:** Yolani de Jesús Bojórquez-Armenta, Luis Gerardo Sarmiento-López, María J. Pozo, Claudia Castro-Martínez, Melina Lopez-Meyer

**Affiliations:** 1 Departamento de Biotecnología Agrícola, CIIDIR-Unidad Sinaloa, Instituto Politécnico Nacional, Guasave 81101, Sinaloa, Mexico; yolaniba09@gmail.com (Y.d.J.B.-A.); clcastro@ipn.mx (C.C.-M.); 2 Unidad de Investigación en Ambiente y Salud, Universidad Autónoma de Occidente Unidad Regional Los Mochis, Los Mochis 81223, Sinaloa, Mexico; luis.sarmiento@uadeo.mx; 3 Department of Plant and Soil Microbiology, Estación Experimental del Zaidín, Consejo Superior de Investigaciones Científicas (CSIC), 18008 Granada, Spain; mariajose.pozo@eez.csic.es

**Keywords:** arbuscular mycorrhiza symbiosis, plant cell wall, endoglucanase, gene expression, *Solanum lycopersicum*, fungal pathogen infection

## Abstract

In this study, we report bioinformatics analysis of the endoglucanase GH9 gene family in tomato (*Solanum lycopersicum* L.) using the SL5.0 genome, confirming the presence of 19 members that clustered into classes A, B, and C. To explore their potential role in plant–microbe interactions, we determined the transcriptional regulation of 10 *SlGH9* gene members in tomato leaves and roots during interactions with the mutualistic root mycorrhizal fungus *Rhizophagus irregularis* and the foliar pathogen *Sclerotinia sclerotiorum*. The upregulation of several *SlGH9* genes in the leaves of mycorrhizal plants suggests that they are involved in cellulose remodeling and biosynthesis rather than its degradation. This would be consistent with the observed increase in foliar area. On the other hand, downregulation of some *SlGH9* genes in leaves of pathogen-infected mycorrhizal plants suggests that these genes may play a role in the enhanced resistance observed by reducing cellulose degradation, thereby maintaining cell wall integrity. The potential involvement of endoglucanase genes in expansive growth (foliar area) and in defense in mycorrhizal and pathogen-infected plants may reflect a growth–defense trade-off.

## 1. Introduction

The plant cell wall is an important cell component that confers crucial features to plants involving morphogenesis and growth, and provides mechanical support for the plant body. Since the cell wall is located outside the protoplast, it is responsive to abiotic stress. It also plays a key role in the plant’s interaction with other microorganisms, mediating communication and serving as a first line of defense against invasion [1,2]. Cell wall-derived components (such as oligosaccharides) induced by abiotic stressors and pathogen infection can be recognized by cell wall receptors as damage-associated molecular patterns (DAMPs), thereby activating the innate immune response [3,4].

Plant cell walls comprise the carbohydrate polymers cellulose, hemicellulose, and pectin. These polymers are complex and diverse, and interact biochemically with other plant cell components, such as lignin, proteins, and suberin, adding complexity and diversity to the plant cell wall [5,6].

Cellulose, the main load-bearing polymer of primary and secondary cell walls [7], is a homopolymer comprising chains of β (1,4) D-glucopyranosyl units that form crystalline structures. Cellulose is synthesized at the plasma membrane by cellulose synthase complexes [8,9]. As cell walls are the primary barrier to potential attackers, phytopathogenic microorganisms have evolved to produce a variety of cell wall-degrading enzymes to degrade plant cell walls as an initial infection mechanism [10]. Endoglucanases (EG) are the first enzymes to act during cell wall degradation and remodeling. They cleave glycosidic bonds in amorphous and crystalline regions of cellulose [11], leaving free ends on the cellulose chain that can be subsequently attacked by cellobiohydrolases and β-glucosidases [12,13,14].

Many studies have focused on microbial endoglucanases, not only because these enzymes act as pathogenic factors in plants, but also because of their potential applications in the processing of lignocellulosic biomass for biofuel production [15]. In addition to endoglucanases produced by microorganisms, plants also produce endogenous ones. Plant endoglucanases are encoded by the glycosyl hydrolase family 9 (GH9) gene family. They may play various roles in cell wall degradation, remodeling, and even biosynthesis across processes such as cell elongation and differentiation, cytokinesis, organ abscission, and fruit ripening [11,16,17]. Plant endoglucanases have a distinctive GH9 catalytic core. They are classified into three classes: A, B, and C. Class A contains an N-terminal cytoplasmic domain, a transmembrane domain, and a C-terminal luminal/extracellular domain with the catalytic domain [18]. Classes B and C have predicted signal peptides at the N-termini for both secretion to the cell wall and the catalytic core. Additionally, class C contains a carbohydrate-binding module at the C-terminal (CBM49), which can bind to crystalline cellulose [11].

Initial studies on endoglucanase gene expression, particularly in tomato (*Solanum lycopersicum* L.), focused on tissue-specific expression and responses to phytohormone treatment. Endoglucanases isolated from ethylene-treated tomato flower abscission zones exhibit differential regulation by the tissue/organ analyzed and by the phytohormones ethylene and auxin [19]. This expression pattern of tomato endoglucanases (*Cel7*) [20], *Cel4* [21], and *Cel5* [22] associates these genes with expansive growth.

In contrast, the transcriptional regulation of plant endoglucanases during biotic interactions is poorly understood. Their role in plant interaction with phytopathogens has been scarcely explored. Tomato endoglucanases *Cel1* and *Cel2* have been reported to be downregulated by necrotrophic fungal infection in the pericarp, at both the mRNA and protein levels [22,23]. Downregulation of *Cel1* and *Cel2* using an antisense approach enhanced resistance to *Botrytis cinerea* infection, suggesting that endoglucanases contribute to susceptibility to pathogens [24].

Even less is known about beneficial plant–microbe interactions, such as arbuscular mycorrhizal symbiosis. Arbuscular mycorrhizal fungi colonize the root cortical cells of most plant species, penetrating plant cell walls to form intracellular symbiotic structures called arbuscules, where exchange between the partners takes place. The interaction yields multiple benefits for the host plant, including enhanced nutrient acquisition, improved resistance to diverse stresses, and increased tolerance to pathogens [25]. As expected, the establishment of this symbiosis causes a complex regulation of gene expression in both symbionts. In tomatoes, some transcriptional studies have been conducted on mycorrhizal plants [26,27,28,29].

It is noteworthy that the regulation of endoglucanases by the AM symbiosis has been studied only in roots. Endoglucanase enzymatic activity in AM colonized roots in lettuce [30] and gene expression in *Medicago truncatula* [31] and *Lotus japonicus* [32] are induced, while their potential regulation in leaves remains unexplored.

On the other hand, recent advances in genome sequencing have demonstrated that endoglucanases (GH9) form multigenic families in multiple plant species. *Arabidopsis* and *Populus* contain more than 20 members, while other species have as few as four (*Fragaria x ananassa* and *Prunus persica*) [11,16,33,34,35]. Tomato also displays a large family, although there are some discrepancies in the proposed number of endoglucanase gene members in this plant species, 24 or 20 *SlGH9* genes, depending on the study [36] and [37], respectively, both using the genome annotation data version SL4.0.

In this study, we present results from a bioinformatic analysis of the GH9 gene family using the genome data version SL5.0 and highlight differences from previous studies using the SL4.0 version. The detailed in silico analysis of the gene family confirmed the presence of 19 members in tomato and provided information on gene and protein structures, as well as their genomic organization. The available data suggest that endoglucanases have diverse functions in multiple essential processes for the plant, including development, growth, and stress responses. Accordingly, their gene expression should likely be tightly regulated depending on the organ and processes in which they are involved. Then, we selected 10 *SlGH9* genes to examine their transcriptional regulation during plant interactions with both beneficial and pathogenic fungi. We hypothesize that endoglucanase genes are downregulated in leaves and pathogen-infected leaves of mycorrhizal plants to reduce cellulose degradation, increase cell wall strength, induce defense priming, and enhance pathogen resistance. In the roots of mycorrhizal plants, we hypothesized that endoglucanases are induced to facilitate cellulose degradation and fungal colonization. To test these hypotheses, we performed qRT-PCR analysis in tomato leaves and roots under mycorrhizal and pathogenic conditions. Our study shed light on the complex regulation of endoglucanases during plant–microbe interactions, both beneficial and pathogenic.

## 2. Results

### 2.1. Identification and Characterization of the Endoglucanase (GH9) Gene Family in Solanum lycopersicum L.

Based on the most recent reference update of the tomato genome (SL5.0), a total of 19 genes in the endoglucanase family (GH9) with the structurally conserved glycoside hydrolase domain were confirmed using InterPro, CDD, and SMART tools. All genes showed the PF00759 (Glyco_hydro_9) domain. A second domain (PF09478) related to carbohydrate-binding module 49 (CBM49) was identified in three of the 19 members. The 19 SlGH9 genes were named *SlGH9-1* to *SlGH9-19*, corresponding to those recently reported by Lin et al. [37]. Gene SlGH9-20 from Lin et al. [37] was excluded from the gene family due to the lack of the conserved active site DAGD. Features of each gene family member, such as gene identification number, chromosomal location, genomic, transcript, coding (CDS), and amino acid sequence lengths, as well as predicted molecular weight (MW) and isoelectric point (PI), are presented in Table 1. Differences in the analysis of this gene family between the SL4.0 and SL5.0 versions of the tomato genome were found and are shown in Table 1, along with the corresponding gene ID in each genome version. Five genes showed differences in the predicted protein lengths between SL4.0 and SL5.0, which explain the differences observed in length, pI, and MW of the corresponding proteins. Also, following these adjustments, the occurrence of signal peptides changed in the genes *SlGH9*-2, *SlGH9*-4, *SlGH9*-8, and *SlGH9*-19 compared with those reported in Lin et al. [37].

Subcellular localization predicted four SlGH9s in the cell membrane, and 14 SlGH9s can be multilocated, both at the cell membrane and secreted at the cell wall. Finally, only SlGH9-18 was predicted to be found at the cell wall. Signal peptide and transmembrane domain prediction indicated that six endoglucanase proteins contain both features (Table S2).

### 2.2. Analysis of Gene Structures, Protein Domains, Conserved Motifs, and Predicted Protein Structures of the SlGH9 Family

The SlGH9 gene family structure was conserved and organized into three protein classes (Figure S1). The glycosyl hydrolase structural domain (GH9) was found in all 19 members, while the CBM49 domain was only in three of them, which corresponds to Class C proteins; Class A is composed of six members, whereas Class B has ten (Figure S1). Structural analysis revealed that the GH9 domain is located at the amino terminus, irrespective of the protein’s class. However, the number and location of introns and exons varied significantly among EG proteins.

**Figure 1:**
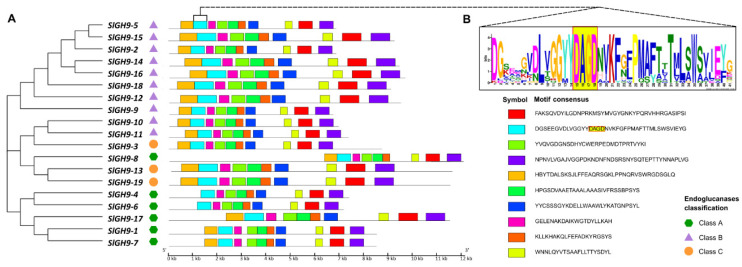
Schematic representation of the conserved protein motifs in tomato endoglucanase (SlGH9) genes. (**A**) Conserved motifs of SlGH9 proteins were identified. Distinctly colored boxes represented ten predicted motifs, and the gray lines indicated non-conserved regions. The sequence logo was created with 19 tomato endoglucanase SlGH9 protein sequences using the MEME program. (**B**) Sequence alignments of the catalytic conserved motif DGSEEGVDLVGGYYDAGDNVKFGFPMAFTTTMLSWSVIEYG in SlGH9 proteins.

The MEME database was used to search for the similarity and diversity of conserved motifs within SlGH9 family members. A total of ten motifs with lengths ranging from 21 to 41 amino acids were identified (Figure 1A). Most SlGH9 proteins contained the ten conserved motifs, except for SlGH9-8, SlGH9-4, and SlGH9-6, all belonging to Class A. Secondary structure analysis reveals that SlGH9 proteins primarily consist of alpha-helical structures (Figure S2). The alignment evidenced the partial presence of the conserved motif DGSEEGVDLVGGYYDAGDNVKFGFPMAFTTTMLSWSVIEYG, the same as in whose amino acid sequence has the DAGD fragment, which is located from amino acid 162 to 165 in all 19 members of the SlGH9 family and serves as a signature amino acid of this group of proteins, and the catalytic site [37] (Figure 1B and Figure S2). The three-dimensional structures of SlGH9 proteins were built from the AlphaFold [38,39] PDB database using homology modeling. As expected, proteins within a class exhibit structural affinities (Figure S3).

### 2.3. Expression Patterns of SlGH9 Genes in Different Tissues

**Figure 2:**
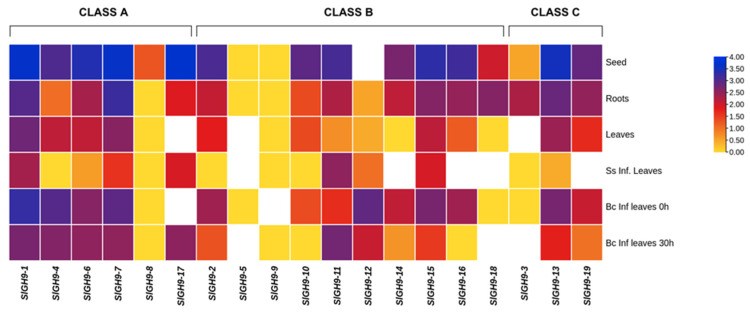
Expression patterns of endoglucanase *SlGH9* genes in different tissues. Heat map representation of RPKM values (Log_2_) for SlGH9 genes in tomato (*Solanum lycopersicum* L.) vegetative tissues (seed, root, leaf, infected leaves with *Sclerotinia Sclerotiorum* (Lib.) DeBary and *Botrytis cinerea* (Pers.) at 0 and 30 h of infection) derived from RNA-seq data (SGN database) for *S. lycopersicum* cv. *Heinz*. White-colored cells indicate actual data values that are Log_2_-normalized to below 0. The color scale represents RPKM Log_2_ values.

The expression profiles of *SlGH9* genes across seeds, roots, and uninfected and infected tomato leaves with two pathogenic fungi, *B. cinerea* and *S. sclerotiorum*, were evaluated using temporal and spatial RNA-seq data retrieved from public datasets (SGN database) and expressed as RPKM values (Log_2_). *SlGH9-5*, *SlGH9-8*, and *SlGH9-9* were expressed at the lowest level in all organs and conditions. In general, most *SlGH9* genes were expressed at higher levels in seeds and roots compared to leaves (Figure 2). No pattern by class was detected in the expression of these genes. However, most *SlGH9* genes were downregulated in leaves infected with pathogenic fungi compared with uninfected leaves, except for *SlGH9-10*, *SlGH9-11*, *SlGH9-12,* and *SlGH9-17*, which showed upregulation (Figure 2).

### 2.4. Chromosome Mapping and SlGH9 Gene Duplication Analysis

Based on the coordinates of *SlGH9* genes annotated in the SL5.0 genome, the physical positions of the SlGH9 family were mapped. Genes were unevenly distributed on tomato chromosomes, with more than half located at or near the telomeric ends. SlGH9 genes were distributed across all chromosomes of the tomato genome, except chromosomes 0 and 10 (Figure S4). Tandem and segmental duplication events were analyzed. These types of duplications are considered the main driving forces behind the expansion of gene families in plant genomes [40]. While no tandem duplication events were identified since the percentages of similarity were lower than 90% (Table S3), six segmental duplication events were identified based on phylogenetic analysis, including the sister pairs *SlGH9-5*/*SlGH9-15*, *SlGH9-14*/*SlGH9-16*, *SlGH9-10*/*SlGH9-11*, *SlGH9-13*/*SlGH9-19*, *SlGH9-4*/*SlGH9-6*, and *SlGH9-1*/*SlGH9-7* (Figure S4 and Table S4). Also, five of six gene pairs exhibited calculated Ka/Ks values less than 1, indicating “negative purification” selection during evolution. Additionally, it was determined that the divergence time occurred between 24 and 63.6 million years ago (Table S4).

### 2.5. Promoter Cis-Acting Regulatory Elements of the S. lycopersicum GH9 Gene Family

**Figure 3:**
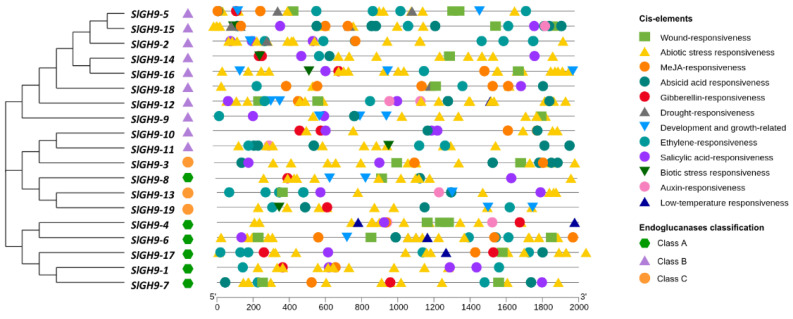
Distribution of *cis*-regulatory elements in the promoter region (2000 bp) of *SlGH9* genes. Different shapes and colors are used to represent regulatory elements, with their respective functions indicated. The PlantCARE tool was used to predict the cis-elements.

To gain a more comprehensive understanding of the putative functional significance of the *SlGH9* gene family, an analysis was conducted on *cis*-regulatory elements located within 2000 bp upstream of the start codon, extracted from the Phytozome database for the 19 gene members. The identified elements were categorized into twelve groups (Figure 3 and Table S5). Promoter regions of the 19 *SlGH9* endoglucanase genes showed different content of *cis*-regulatory elements. The category that presented the highest number of these elements was “abiotic stress” in all three endoglucanase classes (A, B, and C), with the highest *SlGH9-16* (19 elements), and the lowest *SlGH9-18* with four, both from Class B (Table S6). “Drought” and “temperature” presented a low number of *cis*-elements, and most of the *SlGH9* genes did not contain any of these elements. In contrast, phytohormone-responsive elements were more commonly found. “ABA”-responsive elements occur in 16 of the 19 *SlGH9* genes. “MeJA”-responsive elements were also present in 13 out of the 19 members of the family (Table S6), whereas the number of “gibberellin”-responsive elements was generally low. “Ethylene”-responsive elements were also common in the *SlGH9* gene family. Also, 17 out of the 19 *SlGH9* genes contain at least one SA-responsive element. Remarkably, auxin-responsive elements were found in only six genes of the 19 *SlGH9* gene family (Table S6). General patterns in the distribution of cis-element categories in the promoters of the SlGH9 endoglucanase gene family can be identified. Most *SlGH9* genes contain “SA” and “ethylene” responsive elements, but no differences in the average number per gene per class are detected. The presence of phytohormone-responsive elements in *SlGH9* promoters, particularly for MeJA, ABA, SA, gibberellins, and ethylene, is consistent with their roles in growth and development and in plant–microbe interactions.

### 2.6. SlGH9 Gene Expression Profile in Response to Interactions with Beneficial and Pathogenic Fungi

To investigate the potential involvement of *SlGH9* family members in tomato plants’ responses to beneficial and pathogenic interactions. Relative expression levels of 10 selected members from a total of 19 were assessed in the roots and leaves of arbuscular mycorrhizal (M) and non-mycorrhizal (NM) plants, as well as plants challenged with active mycelium of *S. sclerotiorum* on leaves for 24 h using qRT-PCR (NMI and MI). The criterion for selecting these genes was primarily their expression patterns in response to pathogen infection, as inferred from in silico RNA-seq data in Figure 2. The presence of key cis-regulatory elements in their promoter regions, which may be relevant to plant-microorganism interactions, such as symbiosis or pathogenicity, was also revised. This screening strategy allowed us to prioritize genes potentially involved in signaling pathways or stress-responsive networks, thereby enhancing the biological interpretability of the association network analysis of the SlGH9 proteins.

**Figure 4:**
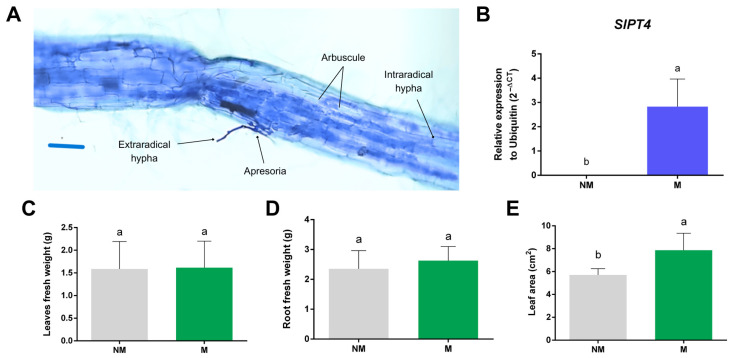
Mycorrhizal colonization of *Solanum lycopersicum* L. roots with *Rhizophagus irregularis*. (**A**) Root segments from mycorrhizal tomato plants were analyzed by light microscopy after trypan blue staining, revealing typical structures of the symbiosis. Scale bar, 100 µm. (**B**) Accumulation of *SlPT4* gene transcripts in *S. lycopersicum* roots under the following treatments: mycorrhizal (M) and non-mycorrhizal (NM). *SlUBQ* (ubiquitin) was used for normalization of qPCR data. Roots (**C**) and leaves (**D**) fresh weight (g). (**E**) Leaf area (cm^2^). Bars represent the means ± standard error of at least three biological replicates. Different letters indicate statistically significant differences, as determined by Student’s *t*-test and Tukey’s post hoc test (*p* ≤ 0.05).

The arbuscular mycorrhizal symbiosis was successfully established, as evidenced by the presence of symbiotic structures, including arbuscules, intraradical and extraradical hyphae (Figure 4A). M plants exhibit 14.2 ± 5.8% colonization. Additionally, the marker gene for mycorrhizal symbiosis functionality, the phosphate transporter 4 (*SlPT4*), showed increased expression in M roots, confirming the establishment and functioning of the symbiosis; no expression of *SlPT4* was found in NM roots (Figure 4B). Plant growth was also measured as fresh weight, and no significant differences were observed between the roots of M and NM plants, nor between the shoots (Figure 4C,D). On the other hand, the effect of mycorrhiza colonization on the foliar area was estimated by comparing the area of the apical leaflet of the third youngest leaves of M vs. NM plants, observing that the foliar area was higher in M than in NM plants (Figure 4E). This is relevant because endoglucanases may be involved in cell expansion.

**Figure 5:**
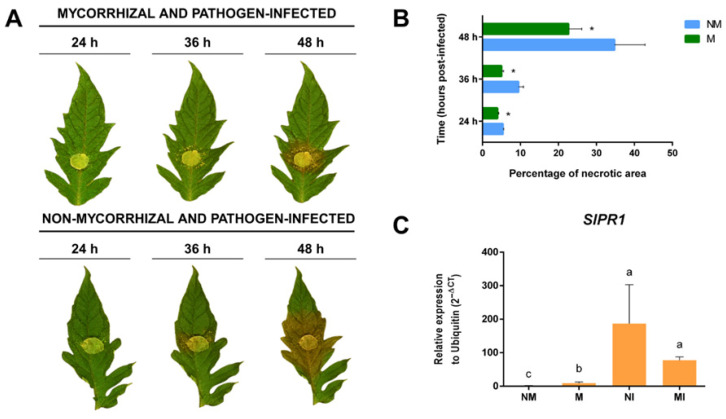
Confirmation of Sclerotinia sclerotiorum infection in tomato *Solanum lycopersicum* (L.) leaves. (**A**) Mycorrhiza-induced resistance to S. sclerotiorum infection in leaves of M vs. NM tomato plants using the detached leaf assay. (**B**) Percentage of necrotic area of S. sclerotiorum infection in leaves of M and NM tomato plants. Student *t*-test and Tukey (*p* ≤ 0.05). Error bars, standard deviation. *, significant differences. (**C**) Relative expression of the SlPR1 was quantified by qRT-PCR to confirm infection by S. lycopersicum in leaves. SlUBQ (ubiquitin) was used as a reference gene. One-way ANOVA and Tukey’s post hoc test (*p* ≤ 0.05). Different letters indicate significant differences.

Plants were infected with *S. sclerotiorum* by placing mycelium plugs on four to five leaflets per plant and allowing the interaction to proceed for 24 h. In most of these leaves, no necrotic lesions were observed after 24 h, although the plant–pathogen interaction had already started. This early time point was selected to avoid potential interference from the pathogen’s endoglucanases once it starts proliferating in the necrotic lesions. To ensure that the interaction of *S. sclerotiorum* mycelium with tomato leaves for 24 h triggered a plant’s defense response, the expression of the gene coding for the pathogenesis-related protein *SlPR1* was determined. As shown in Figure 5C, the expression of this gene was significantly induced in the leaves of infected plants, both M and NM, confirming the onset of the plant’s response to the pathogen.

Mycorrhizal plants are known to show less susceptibility to necrotrophic pathogens compared to non-mycorrhizal plants. To determine if mycorrhiza-induced resistance (MIR) was triggered in mycorrhizal plants, leaves from a different set of M and NM tomato plants were infected with agar plugs containing *S. sclerotiorum* mycelium in a detached leaf assay, and the area of infection was monitored for 48 h. As shown in Figure 5A,B, leaves of M plants exhibit smaller necrotic lesion areas caused by the pathogen, confirming that mycorrhizal plants were efficiently displaying MIR. Additionally, the *SlPR1* gene, associated with pathogen infection, was mainly induced in *S. sclerotiorum*-infected tissues, both in NI and MI (Figure 5C).

**Figure 6:**
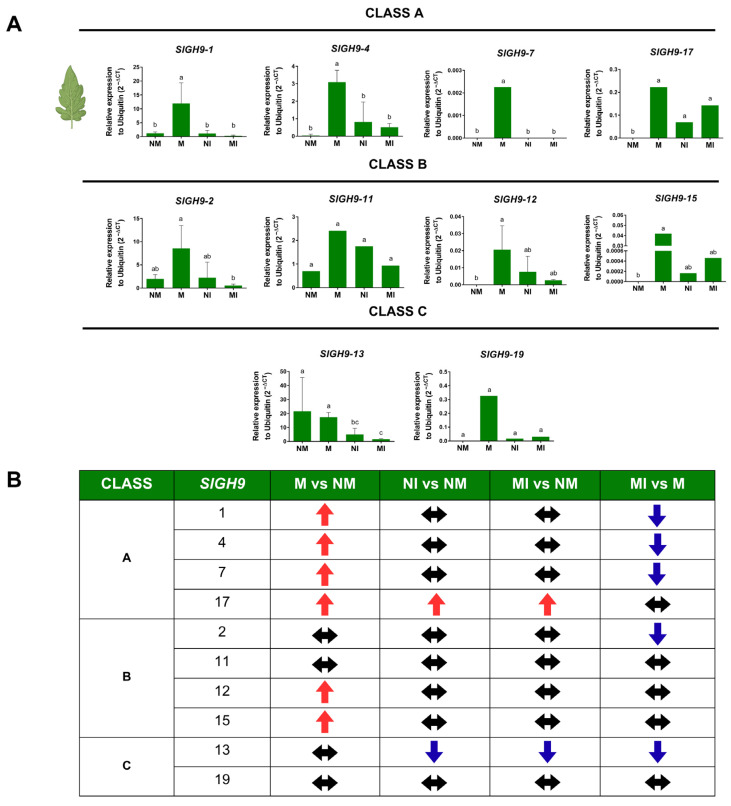
Differential transcript accumulation of *SlGH9* genes in tomato *Solanum lycopersicum* (L.) leaves in response to AM symbiosis and *S. sclerotiorum* infection. (**A**) Relative expression of *SlGH9* genes. (**B**) Summary of the trend in expression per *SlGH9* gene. (↑) Increase in expression relative to NM; (↓) decrease in expression relative to NM; (↔) no change in relative expression. *SlGH9* genes are grouped into Classes A, B, and C. NM: non-mycorrhizal plants; NI: non-mycorrhizal and pathogen-infected plants; M: mycorrhizal plants; MI: mycorrhizal and pathogen-infected plants. Data that met the assumption for parametric analysis were assessed by one-way ANOVA and Tukey’s post hoc test (*p* ≤ 0.05). Error bars represent the means ± standard error of four biological replicates. Different letters indicate statistically significant differences. The data analyzed using the non-parametric Kruskal–Wallis test (*p* ≤ 0.05) are presented in graphs without error bars, and different letters indicate significant differences.

**Figure 7:**
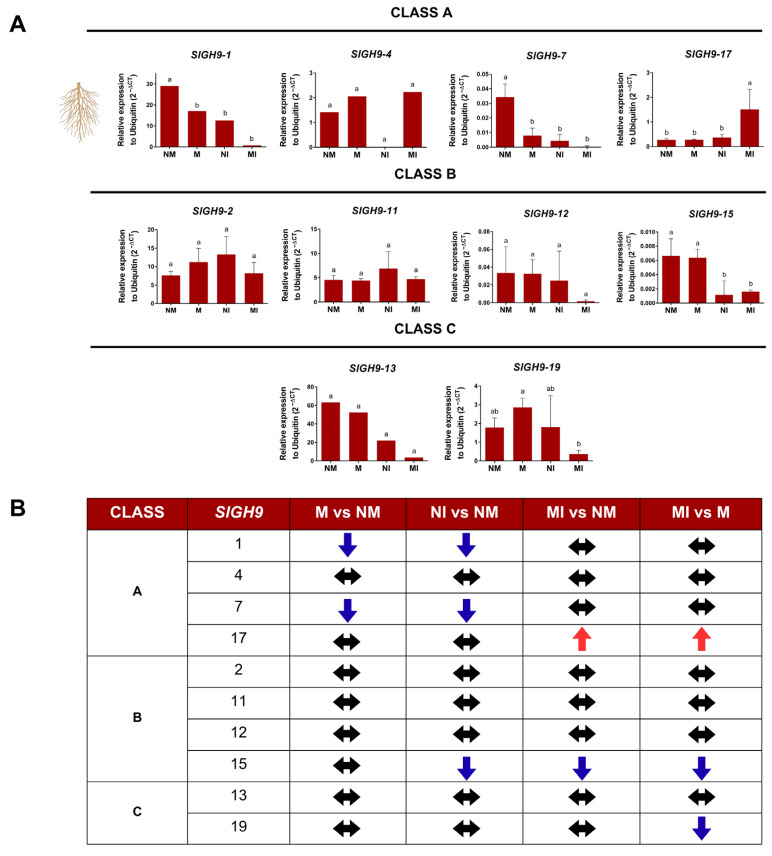
Differential transcript accumulation of SlGH9 genes in tomato *Solanum lycopersicum* (L.) roots in response to AM symbiosis and *S. sclerotiorum* infection. (**A**) Relative expression of SlGH9 genes. (**B**) Summary of the trend in expression per SlGH9 gene in roots. (↑) Increase in expression relative to NM; (↓) decrease in expression relative to NM; (↔) no change in relative expression. *SlGH9* genes are grouped into Classes A, B, and C. NM: non-mycorrhizal plants; NI: non-mycorrhizal and pathogen-infected plants; M: mycorrhizal plants; MI: mycorrhizal and pathogen-infected plants. Data that met the assumption for parametric analysis were assessed by one-way ANOVA and Tukey’s post hoc test (*p* ≤ 0.05). Error bars represent the means ± standard error of four biological replicates. Different letters indicate statistically significant differences. The data analyzed using the non-parametric Kruskal–Wallis test (*p* ≤ 0.05) are presented in graphs without error bars, and different letters indicate significant differences.

The relative expression of the 10 selected *SlGH9* genes was analyzed in leaves and roots of plants from the different treatments, and the results are presented by organ and class in Figure 6 and Figure 7. In leaves, *SlGH9* genes from Class A (*SlGH9-1*, *SlGH9-4*, *SlGH9-7*, *SlGH17*) showed significantly higher expression levels in M plants compared to the NM condition, as well as NI and MI. In contrast, *SlGH9-17* exhibited a distinct profile, with high expression levels in the leaves across the different plant–microbe interactions (M, MI, and NI) and no expression in NM controls (Figure 6A,B). This expression pattern might indicate a special role of SlGH9-17 in plant–microbial interactions. Genes selected from Class B showed less consistent expression profiles. Still, *SlGH9-12* and *SlGH9-15* showed higher expression only in the M condition with respect to NM. Finally, *SlGH9-13* and *SlGH9-19* from Class C exhibited contrasting expression patterns: *SlGH9-13* was the only analyzed endoglucanase *SlGH9* gene to show repression under pathogen infection, regardless of mycorrhizal status, suggesting a specific role during plant interactions with pathogenic microorganisms. *SlGH9-19*, on the other hand, showed no significant changes. Overall, root mycorrhizal colonization induced the expression of most *SlGH9* genes in leaves. In contrast, the foliar pathogen showed a limited impact on their expression; moreover, infection reduced the mycorrhiza-induced expression of several genes.

Gene expression profiles of the endoglucanase *SlGH9* in roots were different from those in leaves, and less consistent changes were found. In contrast to the evident upregulation by mycorrhiza of gene expression in leaves, no induction was observed in the roots of M plants, and even repression was observed in Class A *SlGH9*-1 and *SlGH9*-7. Inoculation with *S. sclerotiorum* in leaves downregulated the expression of *SlGH9*-1, *SlGH9*-7, and *SlGH9*-15 in roots of NI. Interestingly, *SlGH9*-17 was the only gene to show upregulation in roots under the MI condition (Figure 7A,B).

Overall, expression data confirmed differential regulations of *SlGH9* family members during biotic interactions. Surprisingly, mycorrhization had a substantial impact on the transcriptional upregulation of most endoglucanases in leaves. In contrast, the impact on roots was limited to a reduction in the expression levels of two Class A endoglucanases, *SlGH9*-1 and *SlGH9*-7. Pathogen infection had a moderate effect on transcription patterns, but in the dual interaction (MI), the mycorrhiza-induced levels were attenuated, except for *SlGH9*-17.

### 2.7. Association Network Analysis of SlGH9 Protein

An association network analysis of SlGH9 proteins (STRING) revealed a strong association network (PPI enrichment *p* value < 0.05) for eight out of the ten selected SlGH9 proteins; only SlGH9-11 and SlGH9-12 showed enrichment values above the 0.05 threshold, which indicates no association (Figure S5, Table S7). SlGH9-1, SlGH9-4, SlGH9-7, SlGH9-13, and SlGH9-15 exhibited strong interactions with proteins belonging to the glycosyl hydrolase families 1 and 3. SlGH9-2 displayed robust interactions with proteins containing kinase, BHLH, and HECT domains, as well as with proteins from the cullin and β-galactosidase families (Figure S5, Table S7). SlGH9-17, on the other hand, showed a strong interaction with non-specific lipid-transfer proteins, as well as the chitin-binding type 1 domain-containing protein and a protein belonging to the glycosyl hydrolase 1 family. Finally, SlGH9-19 forms a network with a protein containing an F-box domain, a member of the aquaporin family, and proteins belonging to glycosyl hydrolase families 1 and 3. Although the functions of some of the identified interacting proteins are known, as described above, most are uncharacterized (Table S7).

A closer analysis reveals that three selected class A members (SlGH9-1, SlGH9-4, and SlGH9-7) interact with a nearly identical set of proteins (Figure S5, Table S7), consistent with the similar expression patterns of these genes in leaves (Figure 6A). In contrast, the SlGH9-17 interacts with only three common proteins among the other class A members (Figure S6, Table S7) and with seven different proteins. This unique interaction pattern of SlGH9-17 is consistent with its distinct expression pattern in leaves. The class B proteins SlGH9-2, SlGH9-11, SlGH9-12, and SlGH9-15 each have unique sets of interacting proteins, almost exclusively associated with each (Figure S5, Table S7).

### 2.8. Cellobiose and Glucose Content in Tomato Leaves

**Figure 8:**
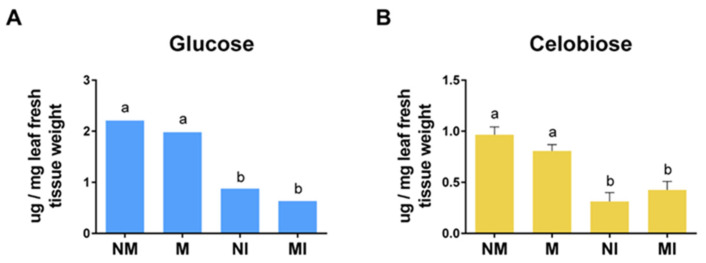
(**A**) Glucose and (**B**) cellobiose content (µg/mg of fresh tissue) in tomato *Solanum lycopersicum* (L.) leaves. NM: non-mycorrhizal plants; NI: non-mycorrhizal and pathogen-infected plants; M: mycorrhizal plants colonized by R. irregularis; MI: mycorrhizal and pathogen-infected plants. Data that met the assumption for parametric analysis were assessed by one-way ANOVA and Tukey’s post hoc test (*p* ≤ 0.05). Error bars represent the means ± standard error of four biological replicates. Different letters indicate statistically significant differences. The data analyzed using the non-parametric Kruskal–Wallis test (*p* ≤ 0.05) are presented in graphs without error bars, and different letters indicate significant differences.

Cellobiose and glucose are the products of the enzymatic degradation of cellulose by endoglucanases; therefore, the pools of these two carbohydrates were determined in leaves of the different treatments. No differences in cellobiose and glucose content were found in leaves of M and NM plants; however, significant differences in their content were detected in infected leaves, regardless of whether they were mycorrhizal (M) or not (NI). No differences were found in the content of cellobiose and glucose between MI and NI roots (Figure 8).

## 3. Discussion

Since plant cell walls are key elements in the interaction of plants with both beneficial and pathogenic microorganisms, some plant endoglucanases might be regulated by plant–microbe interactions. Surprisingly, the regulation and function of these endoglucanases during these interactions are poorly explored. In the present study, the endoglucanase gene family in tomato was reanalyzed, and several members were studied in the context of mycorrhiza colonization and pathogenic fungal infection.

Lin et al. [37] published a genome-wide analysis of the *SlGH9* family using the SL4.0 genome sequence. In this study, the *SlGH9* gene family was characterized bioinformatically using the recently published tomato genome version SL5.0. Some differences were identified in the analysis of this gene family between these two versions of the tomato genome. In SL5.0, five members exhibited different protein lengths, and the signal peptides of several other genes were reassigned. Additionally, one of the previously identified genes (*SlGH9-20*) [37] has been excluded from the family in the present study due to the absence of the protein’s intact active site (DAGD) [37]. Likewise, Luo et al. [36] reported 24 members in the *SlGH9* gene family. Although the extra sequences contain most of the GH9 features required for classification as GH9, they also lack an intact catalytic site. The loss of catalytic site conservation eliminates the protein’s capacity to act as an endoglucanase; however, during evolution, this could have enabled the emergence of carbohydrate-binding enzymes with functions other than endoglucanase. Then, we propose that the *SlGH9* family in tomato now comprises 19 genes, numbered in the present study as *SlGH9*-1 to *SlGH9*-19, as they have been progressively identified across the 12 tomato chromosomes.

Since endogenous plant endoglucanases are involved in the remodeling of the plant cell wall, it is expected that plant–microbe interactions, such as arbuscular mycorrhizal symbiosis and pathogen infection, regulate at least some of the members of the family.

This study focuses on the regulation of *SlGH9* genes in response to a foliar pathogen infection in arbuscular mycorrhiza-colonized plants compared with non-colonized plants. This is because *SlGH9* proteins may play a role in priming a defense response during mycorrhiza colonization or in mycorrhiza-induced resistance (MIR). Since endoglucanase activity is primarily associated with cellulose degradation, we initially hypothesized that downregulating these genes would maintain cellulose integrity and cell wall strength, thereby enhancing resistance to pathogens. In fact, a previous study showed that two tomato endoglucanase genes were downregulated at the RNA level in fruits upon pathogen infection [23], supporting the last hypothesis. Additionally, the in silico expression analysis shown in Figure 2 revealed downregulation of several *SlGH9* members in leaves in response to *S. sclerotiorum*. In contrast, our analysis showed that only one gene (*SlGH9-13*) was downregulated by the pathogen (NI), whereas the rest stayed unchanged compared to the non-infected control (NM). A possible explanation for these discrepancies is differences in the timing and conditions of the pathogen infection assays. In this study, leaf tissues were in contact with *S. sclerotirum* for 24 h. It was confirmed that the plant’s response to the pathogen had initiated, as evidenced by the induction of the defense marker gene PR1; however, necrotic lesions were not yet fully evident. SlGH9-13 may regulate some aspects of cellulose integrity in the plant’s benefit at this early infection stage. The specific role of this gene in leaves of mycorrhizal plants and in pathogen interactions remains to be studied.

On the other hand, five *SlGH9* genes (*SlGH9*-1, *SlGH9*-4, *SlGH9*-7, *SlGH9*-12, and *SlGH9*-15) showed higher expression levels in the leaves of mycorrhizal plants (M), whereas no significant differences were detected in four genes (*SlGH9*-2, *SlGH9*-11, *SlGH9*-13, and *SlGH9*-19). Since the initial hypothesis was that downregulation of endoglucanases would parallel resistance to pathogens by maintaining cellulose integrity and cell wall strength, the induction of *SlGH9* genes in mycorrhizal roots appears contradictory to this hypothesis. However, endoglucanases are not solely involved in cellulose degradation. Still, they can also be associated with cellulose remodeling and even biosynthesis, as endoglucanase activity is required to cleave certain glycosidic bonds during cellulose biosynthesis [41,42,43]. Then, the enhanced expression of some *SlGH9* genes might be related to other responses in M plants, such as leaf growth. In the present study, no differences in leaf growth were observed between M and NM plants; however, a significant difference was detected in foliar area (Figure 4E). Abundant evidence in the literature supports the effect of arbuscular mycorrhizae on the increase in foliar area in tomato [44,45,46], as well as in other plant species. Then, mycorrhiza-induced upregulation of endoglucanases in leaves could modify cellulose rather than degrade it, and the potential impact may be related to increased foliar area and/or growth in M plants. Then, endoglucanases are not involved in the onset of defense priming by mycorrhiza colonization. On the other hand, when leaves of M plants were challenged with *S. sclerotium* (MI), a significant drop in endoglucanase expression was observed in five of the analyzed genes (*SlGH9*-1, *SlGH9*-4, *SlGH9*-7, *SlGH9*-2, and *SlGH9*-13), and the other five genes showed no statistical difference compared with M plants. This downregulation of endoglucanase expression may favor cellulose and cell wall integrity, thereby participating in defense induction.

In other words, endoglucanases may be involved in the increase in foliar area in mycorrhizal plants. At the same time, when a pathogen challenges the leaves of M plants, endoglucanase gene expression is downregulated, possibly halting growth and redirecting resources and energy toward defense. This could represent a growth–defense trade-off. The mechanisms of some growth–defense trade-offs are well recognized and currently under intense study [47].

Regulation of *SlGH9* gene expression by mycorrhiza colonization and foliar pathogen infection appears to be associated with endoglucanase classes. The four class A *SlGH9* genes analyzed showed induction by the symbiotic interaction, and three showed pathogen-mediated repression of this induction, except for *SlGH9*-17. None of the class C endoglucanases were responsive to this interaction. It has been suggested that plant class C endoglucanases are the common ancestor of classes A and B [48,49]. Then, the evolution of endoglucanases may have affected the plant’s response to mycorrhizal symbiosis; however, this remains to be investigated in future work.

*SlGH9-17* expression was induced in all microbial interactions (M, MI, and NMI) compared to the NM condition. Interestingly, *SlGH9*-17 is the only *SlGH9* gene that was not repressed by pathogen infection in leaves of mycorrhiza colonized plants. This gene may be the only *SlGH9* associated with growth even during pathogen infection in MI plant leaves. Its unique protein–protein interaction pattern (Figure S5, Table S7) is consistent with its differential expression patterns compared to other *SlGH9* genes. However, confirming its role in growth in response to mycorrhiza colonization and pathogen infection will require further studies.

Notably, mycorrhiza colonization did not increase the expression of any of the analyzed endoglucanases in M roots compared to NM; in contrast, *SlGH9-1* and *SlGH9-7* exhibited a significant decrease in M roots. Since some degree of reconfiguration of fungal-invaded root cells is needed for mycorrhiza colonization, it is expected that endoglucanase activity is required during colonization. Consistent with this, *LjCel1* from Lotus japonicus was upregulated in mycorrhizal roots [32]. In *Medicago truncatula*, promoter–GUS analysis of transformed roots in composite plants revealed induced expression of an endoglucanase gene (MtCel1) in mycorrhizal roots, specifically associated with arbuscules [50]. A likely ortholog of MtCel1 in tomato, based on sequence similarity, is *SlGH9-17*. Interestingly, in this study, its expression was induced in MI roots but not in M roots.

Network association analysis helps hypothesize or confirm the potential roles of proteins. Most of the proteins predicted to be associated with tomato endoglucanases are currently uncharacterized; however, some information is available for a few of them. *SlGH9*-1, *SlGH9*-4, *SlGH9*-7, *SlGH9*-13, and *SlGH9*-15 interact with proteins belonging to the glycosyl hydrolase families 1 and 3 [51]. This suggests that the mentioned genes are likely associated with cell wall remodeling, consistent with the results obtained in this study. Interestingly, *SlGH9*-17 is associated with two non-specific lipid-transfer proteins, which may play roles in wax or cutin deposition in cell walls and in defense against pathogens. Additionally, the network association analysis links SlGH9-17 to a chitin-binding protein, indicating that it may bind to fungal chitin. This is consistent with the observed upregulation of this gene in *S. sclerotiorum*-infected leaves, supporting the idea that *SlGH9-17* may respond to pathogen infection as part of a defense mechanism. Alternatively, *SlGH9*-17 may promote growth or cell expansion despite pathogen infection. Interestingly, in roots, *SlGH9-17* is the only gene upregulated by the double interaction (MI); thus, it may respond to both pathogen infection and internal mycorrhizal signaling. Although the results presented here suggest that *SlGH9*-17 plays a relevant role in plant responses to plant–microbe interactions, further analysis is required to elucidate its specific role.

In leaves, quantification of free cellobiose and glucose showed no difference in the content of either sugar between NM and M conditions. This is consistent with the idea that the upregulation of endoglucanase genes detected in the leaves of M plants might not be related to pure cellulose degradation, but rather to remodeling cellulose to promote cell growth or expansion. In contrast, the concentration of these sugars was significantly reduced in infected tissues, regardless of the plants’ mycorrhizal status, consistent with previous work [52,53], suggesting that the pathogen, rather than the plants, regulates sugar content in the interaction.

## 4. Materials and Methods

### 4.1. Identification of Endoglucanase Gene Family Members (SlGH9) in Solanum lycopersicum L.

To identify the *SlGH9* gene family in tomato, amino acid and genomic sequences were obtained from the Phytozome v14 database (Walnut Creek, CA, USA; http://www.phytozome.net, accessed on 25 January 2024) and the Sol Genomics Network Ithaca, NY, USA; https://solgenomics.net; accessed on 28 January 2024). To identify all *SlGH9* family members, the endoclucanase-1 gene identifier number (Solyc01g110340) reported previously by Cervantes-Gámez et al. [28] as a cell-wall biogenesis-related gene differentially expressed in tomato leaves of mycorrhizal plants was used as a query sequence to perform a BLASP search in the *S. lycopersicum* genome (SL5.0) in the Phytozome database.

After this initial screening, the Hidden Markov Model (HMM; Cambridgeshire, UK; https://www.ebi.ac.uk/Tools/hmmer/; accessed 1 February 2024) was used to search for profiles of SlGH9 protein domains. All the putative *SlGH9* genes were further confirmed to contain the PF00759 (Glyco_hydro_9) conserved domain using InterPro (Cambridgeshire, UK; https://www.ebi.ac.uk/interpro/, accessed on 3 February 2024), NCBI CD-Search Tool (http://www.ncbi.nln-nih.gov/, accessed on 3 February 2024), and SMART (/) databases.

Genomic, transcriptomic, coding, and peptide sequences, along with chromosomal coordinates, of SlGH9 family members were obtained from the Phytozome v14 database. Molecular and physicochemical properties of SlGH9 proteins, including theoretical isoelectric point (pI) and molecular weight (Mw), were determined using the Compute pI/Mw tool at the ExPASy Swiss bioinformatics resource portal (Lausanne, Switzerland; https://www.expasy.org/, accessed on 5 February 2024). SignalP 6.0 (Lyngby, Denmark; https://services.healthtech.dtu.dk/services/SignalP-6.0/, accessed on 5 February 2024) and DeepTMHMM (Lyngby, Denmark; https://dtu.biolib.com/DeepTMHMM, accessed on 6 February 2024) were used to predict transmembrane domains and signal peptides. Subcellular localization prediction was carried out using DeepLoc-2.0 (Lyngby, Denmark; https://services.healthtech.dtu.dk/services/DeepLoc-2.0/, accessed on 7 February 2024). Finally, each *SlGH9* gene was named sequentially, from top to bottom, according to its chromosome location.

### 4.2. Gene Structure Analysis, Multiple Sequence Alignment, and Conserved Motif Identification

A multiple sequence alignment of sequences encoding the conserved glycosyl hydrolase family 9 domain was constructed using CLUSTAL W. A phylogenetic tree was generated using the maximum likelihood method and LG with freqs. (+F) model with 1000 bootstrap replicates in MEGA 11.

Based on the genomic and coding DNA (CDS) files, the exon-intron structural patterns were obtained using the Gene Structure Display Server (GSDS 2.0) (Beijing, People’s Republic of China; http://gsds.gao-lab.org/, accessed on 12 February 2024). The conserved motifs of *SlGH9* genes were identified via the online tool MEME v 5.5.5 (Seattle, WA, USA; https://meme-suite.org/meme/tools/meme, accessed on 13 February 2024); the number of motifs was set to 10, and other parameters were default. The consensus sequence was analyzed to predict the conserved catalytic motif (DADG) in SlGH9 proteins, which is responsible for catalytic activity [37]. The protein sequence logo was created using the MEME tool.

### 4.3. Sequence Alignment and 3D Structure Prediction of SlGH9 Proteins

The sequence alignment of SlGH9 proteins was performed with Clustal W, and ESPript 3.0 (Lyon, France; https://espript.ibcp.fr/ESPript/ESPript/, accessed on 19 February 2024) was used to predict secondary structures. Three-dimensional (3D) structure was predicted through an analysis of Sequence Similarity Search (SSS) in the FASTA suite program of EMBL-EBI (Cambridgeshire, UK; https://www.ebi.ac.uk/jdispatcher/sss/fasta, accessed on 28 February 2024), and the homology modeling was made with the AlphaFold PDB database (Cambridgeshire, UK; https://alphafold.ebi.ac.uk/, accessed on 21 February 2024).

### 4.4. Chromosome Distribution, Gene Duplication Events, and Analysis of Ka/Ks Ratios of SlGH9 Genes

Gene structure and chromosome information were extracted from the Phytozome v13 database and displayed as a graphical representation of physical location and relative distances using TBtools-II (v.2.326) [54]. Gene duplication events were plotted using Advanced Circos [55]. To analyze gene duplication events, tandem and segmental duplications were considered. A gene pair on the same chromosome located five or fewer gene loci apart and showing more than 90% sequence similarity through an analysis in the EMBOSS Water program using the Smith–Waterman algorithm (Cambridgeshire, UK; https://www.ebi.ac.uk/jdispatcher/psa/emboss_water, accessed on 22 February 2024) [56] was considered a putative tandem duplication. Segmental duplication events were considered for gene pairs located on different chromosomes but clustered in pairs in the phylogenetic tree. Gene selection pressure analysis was conducted using TBtools-II to estimate the synonymous (Ks) and non-synonymous (Ka) substitution rates. In addition, the divergence time of the duplicated genes was calculated using the equation T = Ks/2λ × 10^−6^ million years ago (Mya) for each gene pair, where λ = 1.5 × 10^−8^ substitutions per site per year for dicot plants [57].

### 4.5. Analysis of Cis-Acting Regulatory Elements of Tomato GH9 Genes

The upstream sequences (2.0 Kb) of the start codon of each *SlGH9* family gene were extracted from the Phytozome v14 database as promoter sequences. The PlantCARE tool (Gent, Belgium; http://bioinformatics.psb.ugent.be/webtools/plantcare/html/, accessed on 26 February 2024) [58] was used to further predict cis-elements in the extracted sequences. Finally, this information was mapped using TBtools-II.

### 4.6. In Silico Gene Expression Analysis

To explore the expression pattern of *SlGH9* genes, RNA-seq expression data for *S. lycopersicum* organs including seeds, leaves (healthy and infected with *S. sclerotiorum* and *B. cinerea*), roots, shoots and flowers were downloaded from the GEO database at NCBI (Bethesda, MD; http://www.ncbi.nlm.nih.gov/geo/, accessed on 4 of March 2024) and the Solanaceae crops genome database (SRA049915: accession numbers SRX118613, SRX118614, SRX118615, SRX118616) (FDR less than 3%) [59]. Expression levels were estimated using the ‘reads per kilobase of exon per million fragments mapped’ (RPKM) method and normalized (Log_2_) to examine differences in gene expression across samples. The used datasets provide tissue- and condition-specific expression values, enabling the reconstruction of *SlGH9* gene transcription data with high confidence. To address the limitations of RPKM for cross-sample comparison and to facilitate alignment with our RT-qPCR data, RPKM values were Log_2_-transformed for each tissue and condition. The Log_2_ transformation stabilizes variance, reduces skewness in the data distribution, and enables relative comparisons of expression trends across tissues and treatments—a commonly applied approach in transcriptomic analyses to enhance interpretability. The results were visualized as a heatmap using TBtools-II.

### 4.7. Protein–Protein Interaction Network

The STRING v12 database (Heidelberg, Germany; https://string-db.org, accessed on 6 of March 2024) was used to predict the protein–protein interaction (PPI) network between the selected members of the SlGH9 family and other proteins, based on protein sequences, using default parameters. The PPI network was downloaded and visualized in Cytoscape (v3.10.3). (San Diego, CA, USA; https://cytoscape.org, accessed on 6 of March 2024) [60].

### 4.8. Plant and Fungal Materials and Bioassays

To analyze transcriptional regulation during plant–fungal interactions, bioassays were performed to assess the interaction between tomato plants and the beneficial mycorrhizal fungus *R. irregularis* and the pathogenic fungus *S. sclerotium*.

To acquire plant tissue samples, *S. lycopersicum* (var. Missouri) seeds were surface-sterilized and sown in germination trays containing a mixture of sterilized vermiculite and sand (3:1 *v*/*v*). The trays were maintained at 25 °C in a growth room. For mycorrhizal treatments, after four weeks of growth, the tomato plants were individually transplanted into 0.5 L pots containing the same substrate. At this stage, 400 spores of *R. irregularis* (DAOM 197198) were evenly distributed on the root system of each plant in the mycorrhizal colonized (M) treatment group. AM spores were obtained from a sterile carrot root culture colonized with *R. irregularis* and extracted using the procedure outlined by Cervantes-Gámez et al. [28]. Control plants (NM), which were not colonized, comprised plants mock-inoculated with the final rinse of the spore inoculum wash. Plants were grown in a mixture of sterilized vermiculite and sand (3:1 *v*/*v*) and maintained in a growth room at 25 °C under a 16 h light/8 h dark cycle (250 μmol·m^−2^·s^−1^), 60% humidity. Irrigation was provided once a week with distilled water and twice a week with 30 mL of half-strength Hoagland nutrient solution [61], adjusted to a final phosphate concentration of 50 µM KH_2_PO_4_ to promote mycorrhizal colonization. The NM and M treatment groups’ plants were harvested eight weeks after inoculation with *R. irregularis*. Four biological replicates (plants) for each condition were evaluated.

Sclerotia of *S. sclerotiorum* were collected in common bean agricultural fields in Northern Sinaloa in February 2022. They were surface-sterilized, incubated, and grown in PDA media at 19 °C. For pathogen inoculation of plants, a 0.3 cm diameter agar disk (PDA medium) with *S. sclerotiorum* mycelium from the active growth zone was placed on each leaflet of the mycorrhizal (MI) and non-mycorrhizal (NI) plants, which were then placed in humid chambers to allow infection for 24 h. Then, leaf tissues and immediately after root tissues were harvested, frozen in liquid nitrogen, and stored at −80° C for subsequent molecular analyses. As infection controls, agar plugs without mycelium were placed on tomato leaves.

To assess mycorrhiza-induced resistance, a detached-leaf pathogen bioassay was performed. A leaflet of each M and NM plant was excised and placed in a Petri dish with a wet sterile paper towel at the bottom, and a glass slide was placed on top of the paper to avoid direct contact of the plant tissue with the damp paper. A 0.3 cm diameter agar disk with *S. sclerotiorum* mycelium from the active growth zone was placed on top of the leaflet. The Petri dish was closed, sealed to form a humid chamber, and incubated at 19 °C, and lesion areas were monitored for 48 h. The images were processed in ImageJ (v1.54) (1 cm scale, 8-bit, threshold adjustment), and leaf and lesion areas were calculated using the Analyze and Measure functions. The resulting lesion area percentages obtained were normalized for statistical analysis.

For evaluation of mycorrhizal colonization in roots, half of the root system was separated at harvest, preserved in 50% ethanol for at least overnight, then clarified in 20% KOH, neutralized in 0.1 M HCl, and stained using 0.05% trypan blue in lactoglycerol [62]. Roots of each treatment were maintained in lactoglycerol 1:1:1 (water/lactic acid/glycerol), and total root length colonized by the mycorrhizal fungus was calculated using the line-intersection method [63]. Briefly, after root staining, about fifty 1.5 cm long root segments were randomly selected and set onto a slide, and at least 150 intersection points for observation of fungal structures were registered. The percentage of colonization was calculated using the formula: (number of fungal structures/total number of intersections) * 100.

### 4.9. cDNA Synthesis, Primer Design, and Quantitative RT-PCR Analysis

Total RNA was isolated from the leaves and roots of mycorrhizal colonized (M), non-mycorrhizal (NM), as well as mycorrhizal colonized and *S. sclerotiorum* infected (MI), and non-mycorrhizal and infected (NI) using TRIzol reagent (Invitrogen, Carlsbad, CA), according to the manufacturer’s instructions. For cDNA synthesis, 1 µg of total RNA was used. Oligonucleotide concentrations were tested from 200 to 500 nM to achieve at least 90% efficiency per oligonucleotide pair [28].

Oligonucleotides (primers) for qRT-PCR were designed for the selected *SlGH9* genes based on sequences obtained from the Phytozome v14 database, considering untranslated regions (UTRs) and avoiding the homologous coding regions (CDS). Primer selection parameters such as melting temperature (Tm) and GC content were calculated using OligoCalc (Chicago, IL, USA; https://oligocalc.eu/, accessed on 4 April 2024). To avoid primer dimers or non-specific amplification, the primers for each *SlGH9* gene were tested using temperature-gradient PCR. Primer efficiency was always above 90%. The primers used are listed in Table S1.

Relative quantification of gene expression was performed with SYBR Green (QIAGEN, Germantown, MD, USA) in a QuantStudio™ 7 Flex Real-Time PCR System (Applied Biosystems, Waltham, MA, USA). The qRT-PCR program performed as follows: an initial denaturation at 95 °C for 2 min, followed by 40 cycles of amplification (95 °C for 15 s, annealing/extension at 60 °C for 1 min). Relative expressions of the *SlGH9* genes were calculated using the 2^−ΔCT^ method described by Livak and Schmittgen [64]. Four biological replicates were evaluated for each condition. After confirming low variation among technical replicates, a single technical replicate for each of the four biological replicates was considered. The ubiquitin (*SlUBQ*) gene was used as the reference gene for normalization.

Due to their low expression level in leaves and *S. sclerotiorum*-infected leaves, according to the expression in silico analysis, *SlGH9*-3, *SlGH9*-5, *SlGH9*-8, *SlGH9*-9, *SlGH9*-14, and *SlGH9*-18 were not included in the relative expression quantification. Although SlGH9-10 and SlGH9-16 showed some expression, they were still difficult to measure consistently, so they were not included in the analysis. *SlGH9*-16 presented an interesting expression pattern in the in silico analysis; however, it could not be measured due to technical reasons.

### 4.10. Extraction and Quantification of Sugars in the Leaf Tissue of Tomato Plants

Extraction of total sugars from plant material was carried out according to the method of Zeyner et al. [65] with some modifications. Leaves of M and MI plants (and their respective controls) were frozen in liquid nitrogen, ground in a cold mortar, and kept at –70 °C until processing. Between 100 and 200 mg of ground leaf tissues were added with 1 mL of distilled water and shaken at 100 rpm for 1 h at room temperature. Subsequently, the samples were centrifuged at 14,000 rpm for 5 min. Then, 700 µL of the supernatant was transferred to a new microtube, filtered through a nitrocellulose filter (45 µm), and stored in 2 mL vials at –20 °C until processing.

The total sugar content (cellobiose and glucose) of the extracts was determined using an ultrahigh-performance liquid chromatography (UPLC) equipment (ACQUITY UPCL H-Class PLUS Bio, Waters Milford, MA, USA) with an ion exclusion column Aminex HPX-87H (Bio-Rad, Hercules, CA, USA) using a refractive index detector at 50 °C. The mobile phase used in this analysis was a solution of H_2_SO_4_ (5 mM) at a flow of 0.6 mL/min at 55 °C. Sample injection was 20 µL. The calculations for the sugar molecules evaluated in each sample were obtained by transforming the previously standardized retention time–area values for each sugar molecule. Sugar content in samples was expressed in µg of sugar per µg fresh tissue weight.

### 4.11. Data Analysis

Significant differences in the relative expression levels of *SlGH9-1* to *19*, *SlPT4*, and *SlPR1* genes under the four experimental conditions were assessed using one-way ANOVA followed by Tukey’s post hoc test (α ≤ 0.05), or the non-parametric Kruskal–Wallis test when assumptions for parametric analysis were not met. Normality of the data was evaluated using the Shapiro–Wilk test, and homogeneity of variances was assessed with Levene’s test before statistical analysis. Statistical analyses were performed using IBM SPSS Statistics 25, and data visualization was carried out with GraphPad Prism 6.

## 5. Conclusions

A reanalysis of the constitution of the *GH9* endoglucanase gene family in tomato, using the SL5.0 version of the tomato genome, identified 19 genes that contain the distinctive GH9 catalytic core in an intact form. Since plant endoglucanases act on cellulose, the primary component of plant cell walls, we hypothesize that these genes are regulated during plant–microbe interactions, both beneficial and pathogenic. Our results are consistent with the idea that the role of these endoglucanases in leaves of mycorrhizal plants may be more related to cellulose remodeling and biosynthesis than to its degradation. This supports the notion that endoglucanases are involved in the increase in leaf foliar area in mycorrhizal plants rather than in defense. On the other hand, the downregulation of endoglucanases during pathogen infection in leaves of M plants suggests that at least some endoglucanases might be involved in mycorrhiza-induced resistance. All this aligns with the growth–defense trade-off already described in plants.

## Figures and Tables

**Table 1:** Structural features of endoglucanase (GH9) family genes in tomato (*Solanum lycopersicum* L.). Values in bold and marked with an asterisk denote differences between our findings and those reported by Lin et al. [37].

Name	Gene ID		Location		Size							Class	Subcellular Localization
	SL5.0	SL4.0	Chr	Start/End	Genomic	Transcript	5′ UTR	3′ UTR	Protein	PI	MW (KDa)		
*SlGH9-1*	Solyc01G003470	Solyc01g102580	1	86,055,748/86,059,426	3678	2242	107	281	618	8.9	68.5	A	Cell membrane
*SlGH9-2*	Solyc01G004213	Solyc01g110340	1	91,850,248/91,856,033	5785	1491	0	0	497 *	4.9 *	55.2 *	B	Cell membrane/Cell wall
*SlGH9-3*	Solyc02G000158	Solyc02g014220	2	15,089,049/15,093,042	3993	2127	48	180	633	7.6	69.8	C	Cell membrane/Cell wall
*SlGH9-4*	Solyc02G002026	Solyc02g083980	2	47,539,277/47,542,107	2830	1605	0	0	535 *	5.5 *	59.2 *	A	Cell membrane/Cell wall
*SlGH9-5*	Solyc03G001799	Solyc03g083820	3	49,794,157/49,796,385	2228	1497	0	0	499	8.4	55.4	B	Cell membrane/Cell wall
*SlGH9-6*	Solyc04G002891	Solyc04g081300	4	66,400,254/66,405,738	5484	2101	300	241	520	5.6	56.6	A	Cell membrane
*SlGH9-7*	Solyc05G000008	Solyc05g005080	5	144,494/147,193	2699	2231	136	244	617	8.9	68.3	A	Cell membrane
*SlGH9-8*	Solyc05G002376	Solyc05g052530	5	62,595,709/62,607,754	12045	2759	0	140	873 *	7.9 *	99.4 *	A	Cell membrane/Cell wall
*SlGH9-9*	Solyc06G001635	Solyc06g066120	6	43,899,874/43,903,182	3308	1944	110	373	487	8.5	54.1	B	Cell membrane/Cell wall
*SlGH9-10*	Solyc07G001834	Solyc07g049300	7	59,881,351/59,885,115	3764	1862	146	204	504	6.02	57.0	B	Cell membrane/Cell wall
*SlGH9-11*	Solyc07G002653	Solyc07g064870	7	67,299,221/67,303,837	4616	1602	0	0	534	8.7	60.9	B	Cell membrane/Cell wall
*SlGH9-12*	Solyc08G002413	Solyc08g081620	8	66,255,855/66,259,192	3337	1578	72	0	502	7.5	55.2	B	Cell membrane/Cell wall
*SlGH9-13*	Solyc08G002479	Solyc08g082250	8	66,732,363/66,737,321	4958	2196	194	124	626	9.3	68.9	C	Cell membrane/Cell wall
*SlGH9-14*	Solyc08G002569	Solyc08g083210	8	67,444,966/67,447,305	2339	1970	225	251	498	6	54.4	B	Cell membrane/Cell wall
*SlGH9-15*	Solyc09G000424	Solyc09g010210	9	3,689,632/3,694,758	5126	1606	73	63	490	8.4	54.1	B	Cell membrane/Cell wall
*SlGH9-16*	Solyc09G002275	Solyc09g075360	9	63,989,601/63,992,815	3214	1732	72	127	511	9.05	56.2 *	B	Cell membrane/Cell wall
*SlGH9-17*	Solyc11G000365	Solyc11g008820	11	3,075,800/3,082,697	6897	2118	39	171	636 *	9.1 *	71.3 *	A	Cell membrane
*SlGH9-18*	Solyc11G001671	Solyc11g040340	11	40,371,634/40,373,802	2168	1440	0	0	480	8.7	53.4	B	Cell wall
*SlGH9-19*	Solyc12G002184	Solyc12g055970	12	62,863,919/62,868,221	4302	2209	27	328	618 *	6.3 *	68.3 *	C	Cell membrane/Cell wall

## Data Availability

Data are contained within this study.

## Supplementary Materials

The following supporting information can be downloaded at: https://www.mdpi.com/article/10.3390/plants14223458/s1; Table S1. Primers used for RT-qPCR; Table S2. Transmembrane domains and signal peptides prediction of the GH9 family; Table S3. Tandem duplication of sister pair genes of endoglucanases (GH9) in tomato (*Solanum lycopersicum* L.); Table S4. Segmental duplication of endoglucanase (GH9) gene pairs in tomato (*Solanum lycopersicum* L.); Table S5. Regulatory *cis*-elements within 2000 bp upstream of the 19 *SlGH9* genes; Table S6. Number of *cis*-elements in the promoters of *SlGH9* genes per category and class; Table S7. Protein–protein interaction network (PPI) of the ten selected SlGH9; Figure S1. Phylogenetic relationships, gene structure, and conserved domain architecture of the SlGH9 gene family in tomato; Figure S2. Secondary structure of SlGH9 family protein; Figure S3. Tertiary structure prediction of SlGH9 proteins; Figure S4. Chromosomal locations of *SlGH9* genes; Figure S5. Interaction network analysis; Figure S6. Interacting proteins of the selected SlGH9 proteins.
