# Supplementary material for: Characterization of Endoglucanase (GH9) Gene Family in Tomato and Its Expression in Response to Rhizophagus irregularis and Sclerotinia sclerotiorum
Source: Plants (Basel). 2025 Nov 12;14(22):3458. doi: 10.3390/plants14223458 (PMC12656509; doi:10.3390/plants14223458)
Supplement: Supplementary file 1 [file plants-14-03458-s001.zip › plants-3927842-supplementary.pdf]

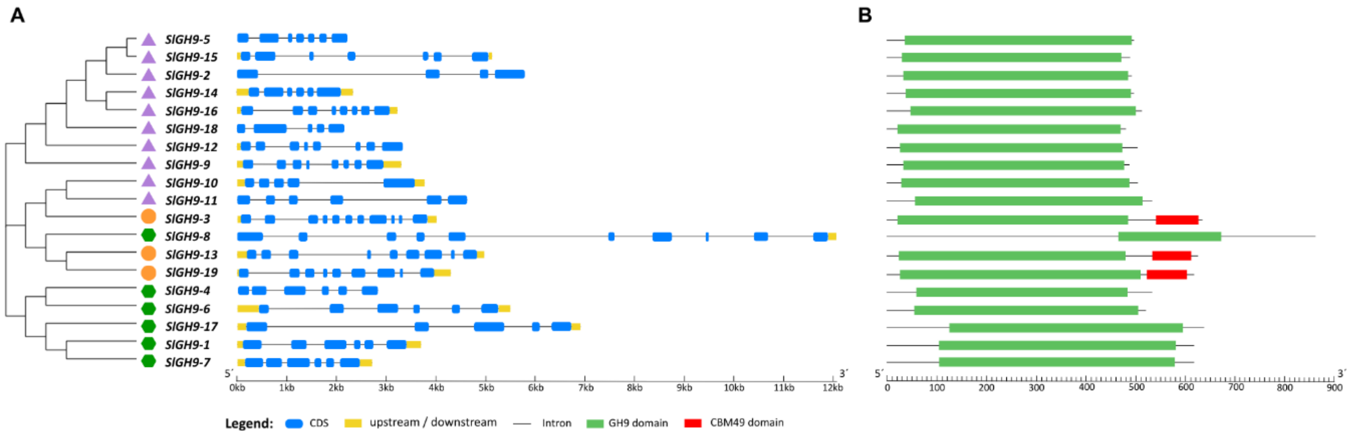

Figure S1. Phylogenetic relationships, gene structure, and conserved domain architecture of the SlGH9 gene family in tomato. A) The tree was constructed using the maximum likelihood LG +F model, grouping genes into three clades and three protein-based classes (green hexagons: Class A; purple triangles: Class B; orange circles: Class C). Gene structure diagrams illustrate the organization of exons, introns, and UTRs. B) Conserved domains GH9 and CBM49 are depicted as light green and red rectangles, respectively. Conserved domains are shown in nucleotide coordinates.

Figure S2. Secondary structure of SlGH9 family protein.

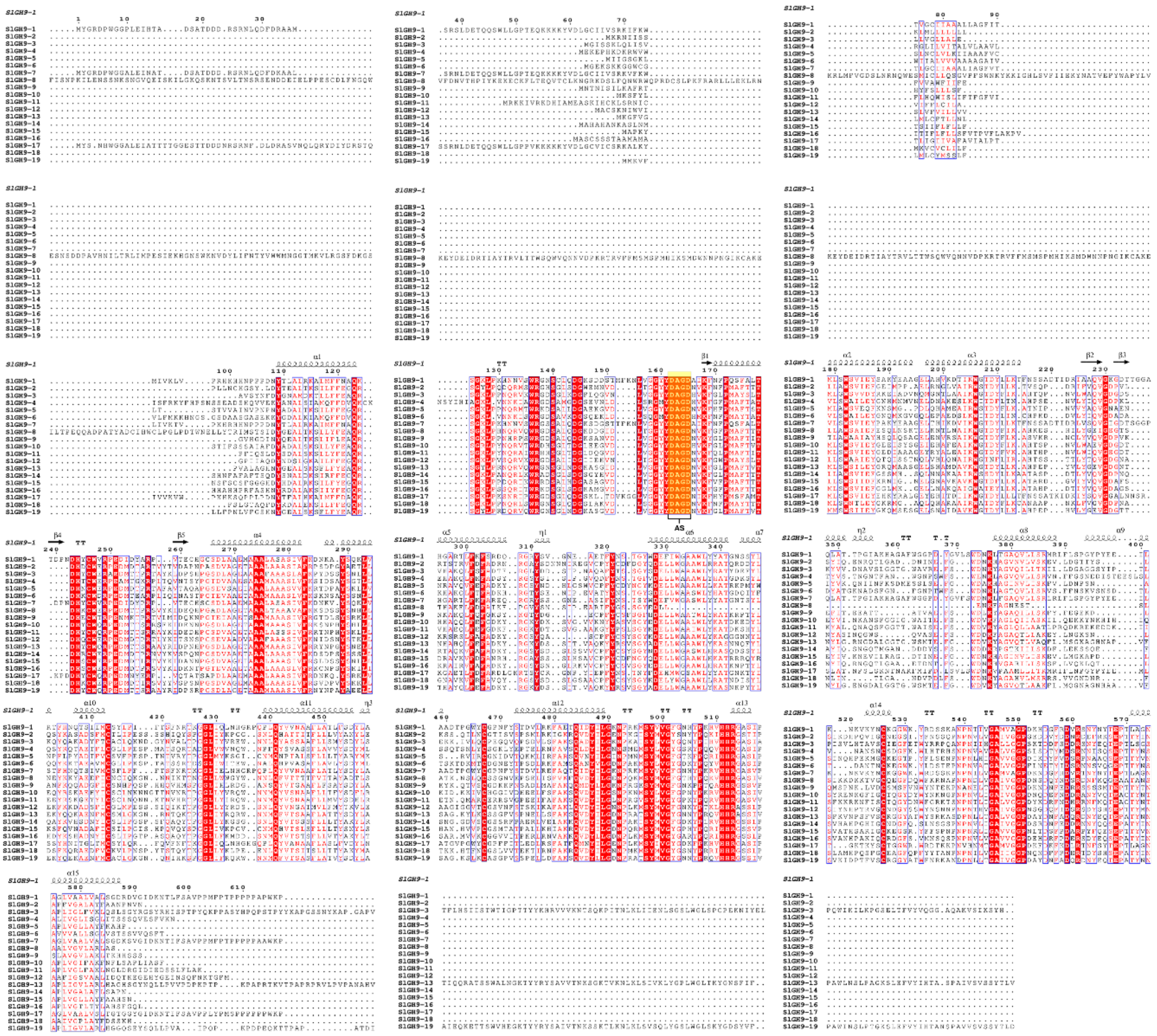

Figure S3. Tertiary structure prediction of SlGH9 proteins.

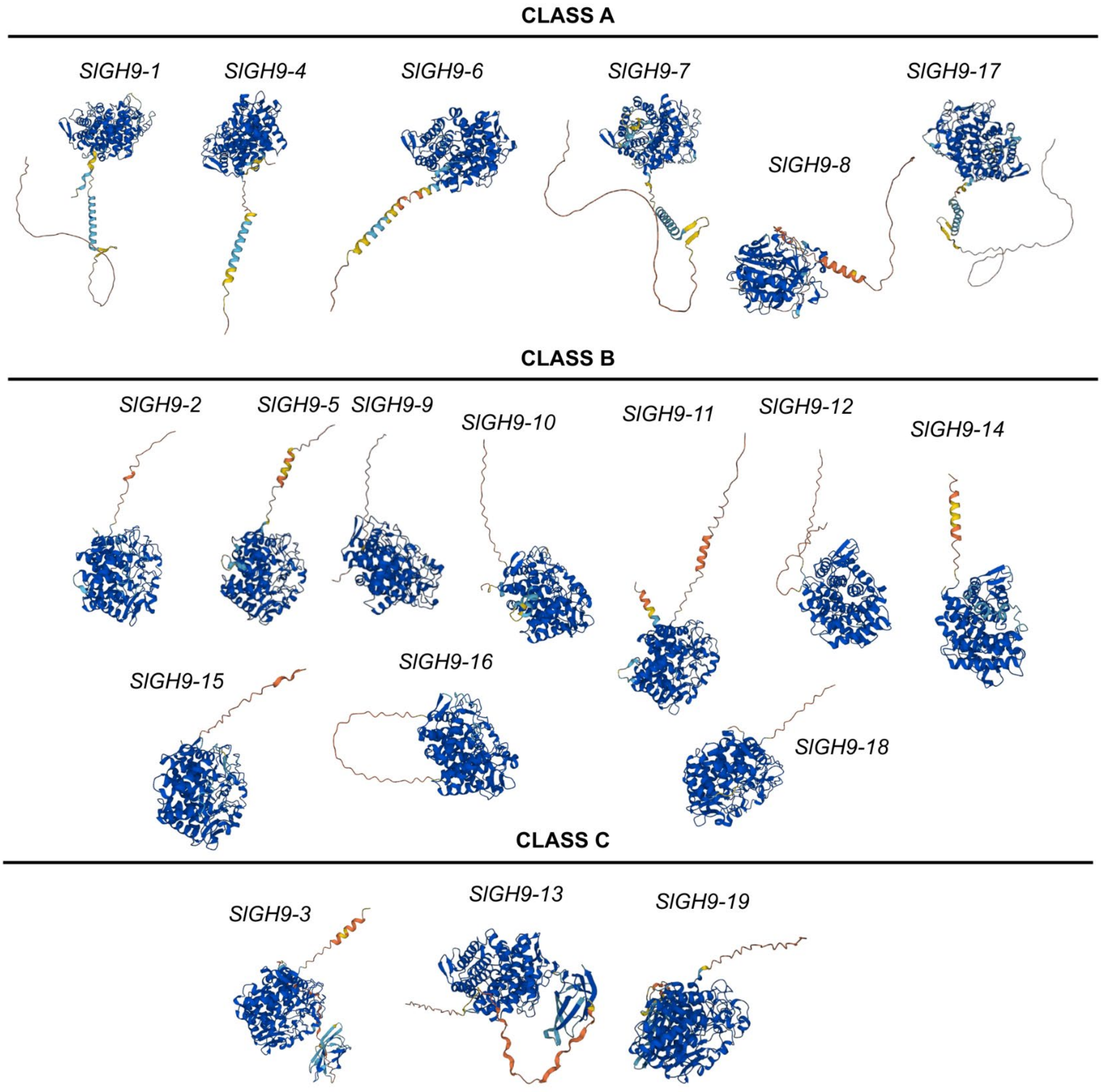

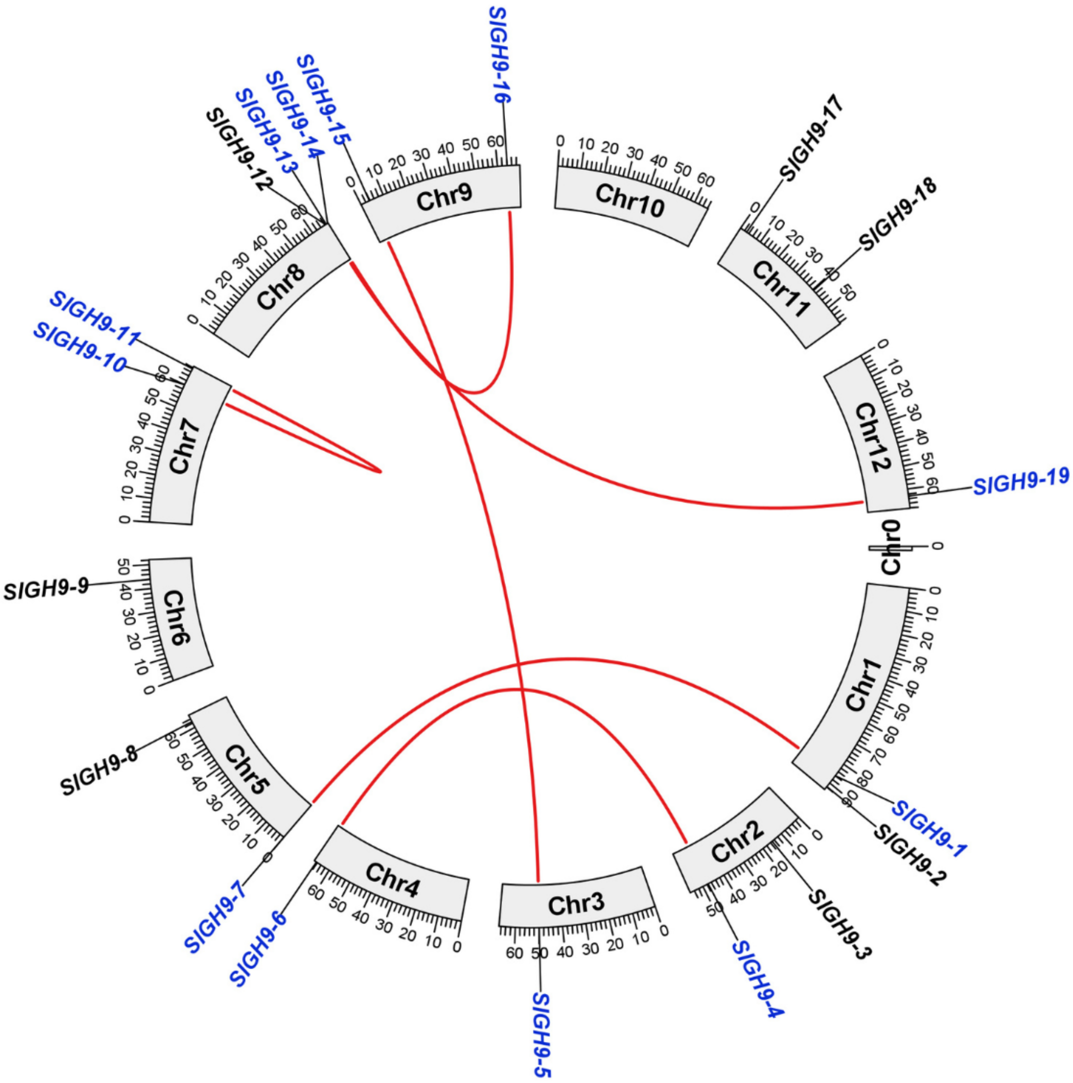

Figure S4. Chromosomal locations of *SlGH9* genes. Red connecting lines and blue gene labels indicate gene pairs arising from segmental duplications.

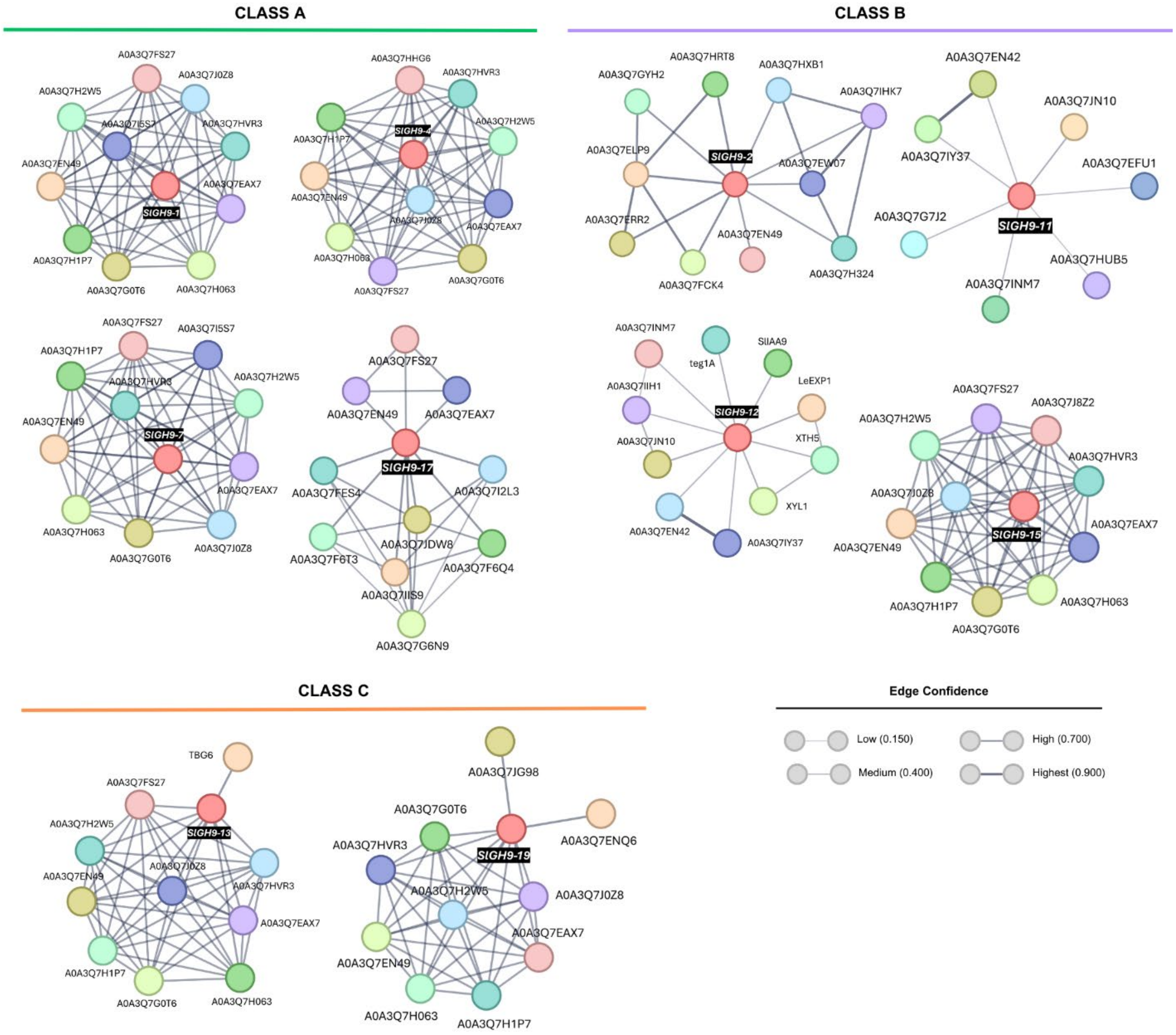

Figure S5. Interaction network analysis. Network nodes denote proteins, and edges represent the presence of an interaction. The edge thickness indicates the strength of data support, while the red nodes specifically indicate members of the SlGH9 protein family.

Figure S6. Interacting proteins of the selected SlGH9 proteins**.**

Interacting protein	Function	A				B				C	
		SlGH9-1	SlGH9-4	SlGH9-7	SlGH9-17	SlGH9-2	SlGH9-11	SlGH9-12	SlGH9-15	SlGH9-13	SlGH9-19
A0A3Q7EN49	Uncharacterized protein.	■	■	■	■	■			■	■	■
A0A3Q7GOT6	Uncharacterized protein.	■	■	■					■	■	■
A0A3Q7H063	Uncharacterized protein.	■	■	■					■	■	■
A0A3Q7H1P7	Uncharacterized protein; Belongs to the glycosyl hydrolase 3 family	■	■	■					■	■	■
A0A3Q7H2W5	Uncharacterized protein.	■	■	■					■	■	■
A0A3Q7HVR3	Uncharacterized protein.	■	■	■					■	■	■
A0A3Q7J0Z8	Uncharacterized protein.	■	■	■					■	■	■
A0A3Q7I5S7	Uncharacterized protein.	■		■							
A0A3Q7EAX7	Uncharacterized protein; Belongs to the glycosyl hydrolase 1 family	■	■	■	■				■	■	■
A0A3Q7FS27	Uncharacterized protein.	■	■	■	■				■	■	
A0A3Q7HHG6	Uncharacterized protein; Belongs to the glycosyl hydrolase 1 family.		■								
A0A3Q7IIS9	Non-specific lipid-transfer protein				■						
A0A3Q7JDW8A	Non-specific lipid-transfer protein				■						
A0A3Q7G6N9	Chitin-binding type-1 domain-containing protein				■						
A0A3Q7F6Q4	Uncharacterized protein.				■						
A0A3Q7F6T3	Uncharacterized protein.				■						
A0A3Q7FES4	Uncharacterized protein.				■						
A0A3Q7I2L3	Uncharacterized protein.				■						
A0A3Q7ELP9	Protein kinase domain-containing protein					■					
A0A3Q7ERR2	Uncharacterized protein.					■					
A0A3Q7EW07	Beta-galactosidase; Belongs to the glycosyl hydrolase 35 family.					■					
A0A3Q7FCK4	BHLH domain-containing protein.					■					
A0A3Q7GYH2	BHLH domain-containing protein.					■					

A0A3Q7H324	CULLIN_2 domain-containing protein; Belongs to the cullin family.										
A0A3Q7HRT8	BHLH domain-containing protein.										
A0A3Q7HXB1	CULLIN_2 domain-containing protein; Belongs to the cullin family.										
A0A3Q7IHK7	HECT domain-containing protein.										
A0A3Q7EFU1	Dihydrolipoamide acetyltransferase component of pyruvate dehydrogenase complex.										
A0A3Q7EN42	WD_REPEATS_REGION domain-containing protein.										
A0A3Q7G7J2	Uncharacterized protein.										
A0A3Q7HUB5	Uncharacterized protein; Belongs to the WD repeat SEC13 family.										
A0A3Q7INM7	Protein kinase domain-containing protein; Belongs to the protein kinase superfamily.										
A0A3Q7IY37	B30.2/SPRY domain-containing protein.										
A0A3Q7JN10	Uncharacterized protein.										
LeEXP1	Expansin										
XYL1	Fn3_like domain-containing protein.										
SlIAA9	Auxin-responsive protein.										
XTH5	GH16 domain-containing protein.										
teg1A	Beta-galactosidase.										
A0A3Q7IY37	B30.2/SPRY domain-containing protein.										
A0A3Q7IIH1	Cellulase domain-containing protein.										
A0A3Q7INM7	Protein kinase domain-containing protein.										
TBG6	Beta-galactosidase										
A0A3Q7J8Z2	Uncharacterized protein; Belongs to the glycosyl hydrolase 1 family.										
A0A3Q7ENQ6	F-box domain-containing protein.										
A0A3Q7JG98	Uncharacterized protein; Belongs to the MIP/aquaporin (TC 1.A.8) family.										

Table S1. Primers used for RT-qPCR

Gene	Forward (5' – 3')	Reverse (5' – 3')
SlGH9-1	TCC TTC CTC CAT AAT ACC CC	ACCGTACATATTGATTCTGGG
SlGH9-2	TGGAAGCAATTTACAACAT	CCGCGAATGATGAATTTTTTG
SlGH9-4	ATCCGTCTAATTCTTCAGAGGC	CCGAATCTCCTCTCCAAGCA
SlGH9-7	TTG TTT GTT GGT GAA TGA AGG G	CCGACACTACTAGACAAACAT
SlGH9-11	GACGTAAAACCTCGAACCCG	CGATTCCTCTGTCTAGCCC
SlGH9-12	AGAAGGAGAACACTATGGGG	ATATGACAAGTGAAAAGGACGG
SlGH9-13	ATA AAC TAA CCA GCA TTG CCC	CCCAGCTAATGCTACTGGA
SlGH9-15	CAAATTTAACAGACTCGTTCCC	GCCTTTGCACACCACTACAT
SlGH9-17	AGTACAGAGCCCTTGGTGAA	TGTTCAAAGCACCACCTACC
SlGH9-19	ATCAAAGAGTTCAATGGAGGGG	CCCATACTCAATTATGCTCCAT
SlUBQ	ACCAAGCCAAAGATCAAGC	GTGAGCCCACACTTACCACAGT
SlPT4	GAAGGGGAGCCATTTAATGTGG	ATCGCGGCTTGTTTAGCATTTCC
SlPR1	GCAACAACGGGTGGTACTTT	ATGGACGTTGTCCTCTCCAG

Table S2. **Transmembrane domains and signal peptides prediction of the GH9 family.** SP, signal peptide; TM, transmembrane domain; Inside/Membrane, number of amino acids located inside/out of the membrane.

Gene	SP	TM	Inside	Membrane	Outside
SlGH9-1	–	A1	1 – 70	71 – 92	93 – 617
SlGH9-2	1 – 25	0	–	–	26 – 496
SlGH9-3	1 – 24	C0	–	–	25 – 632
SlGH9-4	-	A1	1 – 11	12 – 32	33 – 534
***SlGH9**-5*	**1 – 31**	0	–	–	32 – 498
SlGH9-6	–	A1	1 – 10	11 – 31	32 – 519
SlGH9-7	–	A1	1 – 70	71 – 92	93 – 616
***SlGH9**-8*	**–**	A1	1 – 33	34 – 49	50 – 872
***SlGH9**-9*	**1 – 27**	0	–	–	28 – 486
SlGH9-10	1 – 25	0	–	–	26 – 503
SlGH9-11	**1 – 48**	0	–	–	49 – 533
SlGH9-12	1 – 24	0	–	–	25 – 501
SlGH9-13	1 – 21	C0	–	–	22 – 625
SlGH9-14	1 – 30	0	–	–	31 – 497
SlGH9-15	1 – 23	0	–	–	24 – 489
SlGH9-16	1 – 30	0	–	–	31 – 510
SlGH9-17	–	A1	1 – 88	89 – 111	112 – 635
SlGH9-18	1 – 26	0	–	–	17 – 479
***SlGH9**-19*	1 – 24	C0	–	–	26 – 617

Table S3. Tandem duplication of sister pair genes of endoglucanases (GH9) in tomato (*Solanum lycopersicum* L.).

Gene pairs	Score	Identity (%)	Similarity (%)
SlGH9-1/*SlGH9-2*	8103.0	38.6	38.6
SlGH9-10/ *SlGH9-11*	9295.0	46.4	46.4
SlGH9-12/ *SlGH9-13*	7786.0	46.6	46.6
SlGH9-12/ *SlGH9-14*	5607.5	43.5	43.5
SlGH9-13 SlGH9-/14	5611.5	39.6	39.6

Table S4. Segmental duplication of endoglucanase (GH9) gene pairs in tomato (*Solanum lycopersicum* L.).

Duplicate genes	Ka	Ks	Ka/Ks	Duplication Date (MY)	Purifying Selection
SlGH9-5 / *SlGH9-15*	0.36518746	NaN	NaN	-	-
SlGH9-14 / *SlGH9-16*	0.325410269	1.703	0.190	56.7	Yes
SlGH9-10 / *SlGH9-11*	0.215429622	1.876	0.114	62.5	Yes
SlGH9-13 / *SlGH9-19*	0.113610819	0.819	0.138	27.3	Yes
SlGH9-4 / *SlGH9-6*	0.284236826	1.909	0.148	63.6	Yes
SlGH9-1 / *SlGH9-7*	0.062266443	0.720	0.086	24.0	Yes

Abbreviations used in table: Ka (nonsynonymous substitutions), Ks (synonymous substitutions), MY (Millions of Years), Ka/Ks<1 is considered as purifying selection for *SlGH9* gene family.

Table S5. Regulatory *cis*-elements within 2000 bp upstream of the 19 *SlGH9* genes.

Gene	Cis-element	Individual	Total	Category
SlGH9-1	STRE	2	10	Abiotic stress responsiveness
	MYB	3		
	MYC	5		
	ERE	2	2	Ethylene-responsiveness
	GARE-motif	1	1	Gibberellin-responsiveness
	CGTCA-motif	1	2	MeJA-responsiveness
	TGACG-motif	1		
	TCA	3	3	Salicylic acid-responsiveness
SlGH9-2	MYB	6	13	Abiotic stress responsiveness
	MYC	5		
	STRE	2		
	ABRE	1	1	Absicid acid responsiveness
	CAT-box	1	1	Development and growth-related
	MBSI	1	1	Drought-responsiveness
	ERE	6	6	Ethylene-responsiveness
	CGTCA-motif	1	2	MeJA-responsiveness
	TGACG-motif	1		
	TCA-element	3	3	Salicylic acid-responsiveness
SlGH9-3	WUN-motif	2	2	Wound-responsiveness
	MYB	2	11	Abiotic stress responsiveness
	MYC	7		
	TC-rich repeats	2		
	ABRE4	3	11	Absicid acid responsiveness
	ABRE3a	3		
	ABRE	5		
	CGTCA-motif	2	2	MeJA-responsiveness
	TCA	2	2	Salicylic acid-responsiveness
SlGH9-4	AuxRR-core	1	2	Auxin-responsiveness

	TGA-element	1		
	MYB	4		
	MYC	6	12	Abiotic stress responsiveness
	STRE	2		
	MBS	1	1	Drought-responsiveness
	CGTCA-motif	1		MeJA-responsiveness
	TGACG-motif	1	2	
	LTR	2	2	Low-temperature responsiveness
	W box	2	4	Wound-responsiveness
	WUN-motif	2		
	TCA-element	1	1	Salicylic acid-responsiveness
	P-box	1	1	Gibberellin-responsiveness
SlGH9-5	W box	2	3	Wound-responsiveness
	WUN-motif	1		
	STRE	1		
	MYC	4	8	Abiotic stress responsiveness
	MYB	3		
	CGTCA-motif	2	4	MeJA-responsiveness
	TGACG-motif	2		
	ABRE	1	1	Absicid acid responsiveness
	P-box	1	1	Gibberellin-responsiveness
	ARE	2	2	Drought-responsiveness
	O2-site	2	2	Development and growth-related
	ERE	4	4	Ethylene-responsiveness
SlGH9-6	MBS	2	2	Drought-responsiveness
	MYB	9		
	MYC	5	15	Abiotic stress responsiveness
	STRE	1		
	TGACG-motif	3	6	MeJA-responsiveness
	CGTCA-motif	3		
	ERE	3	3	Ethylene-responsiveness
	ABRE3a	1		
	ABRE	1	3	Absicid acid responsiveness
	ABRE4	1		
	LTR	1	1	Low-temperature responsiveness
	W box	3	3	Wound-responsiveness

	TCA-element	1	1	Salicylic acid-responsiveness
	AP-1	1	1	Development and growth-related
SlGH9-7	MYB	6	11	Abiotic stress responsiveness
	MYC	4		
	STRE	1		
	GARE-motif	1	1	Gibberellin-responsiveness
	ERE	2	2	Ethylene-responsiveness
	ABRE	3	6	Absicid acid responsiveness
	ABRE2	1		
	ABRE3a	1		
	ABRE4	1		
	CGTCA-motif	1	2	MeJA-responsiveness
	TGACG-motif	1		
	TCA	1	1	Salicylic acid-responsiveness
	WUN-motif	2	2	Wound-responsiveness
SlGH9-8	WUN-motif	1	1	Wound-responsiveness
	ABRE4	1	3	Absicid acid responsiveness
	ABRE3a	1		
	ABRE	1		
	O2-site	1	2	Development and growth-related
	CAT-box	1		
	GARE-motif	1	1	Gibberellin-responsiveness
	TC-rich repeats	1	11	Abiotic stress responsiveness
	STRE	2		
	MYB	1		
	MYC	7		
	TCA-element	1	1	Salicylic acid-responsiveness
SlGH9-9	STRE	3	8	Abiotic stress responsiveness
	MYB	3		
	MYC	1		
	TC-rich repeats	1		
	ABRE3a	1	3	Absicid acid responsiveness
	ABRE4	1		
	ABRE	1		
	O2-site	1	3	Development and growth-related
	CAT-box	2		
	WUN-motif	1	1	Wound-responsiveness

	TCA-element	1	2	Salicylic acid-responsiveness
	TCA	1		
	ERE	1	1	Ethylene-responsiveness
SlGH9-10	TGACG-motif	2	4	MeJA-responsiveness
	CGTCA-motif	2		
	MYB	2	5	Abiotic stress responsiveness
	TC-rich repeats	1		
	MYC	2		
	TCA-element	2	3	Salicylic acid-responsiveness
	TCA	1		
	P-box	1	2	Gibberellin-responsiveness
	TATC-box	1		
	ABRE	2	2	Absicid acid responsiveness
SlGH9-11	STRE	2	11	Abiotic stress responsiveness
	TC-rich repeats	1		
	MYC	3		
	MYB	5		
	ABRE	6	8	Absicid acid responsiveness
	ABRE3a	1		
	ABRE4	1		
	WRE3	1	1	Biotic stress responsiveness
	TGA-element	1	1	Auxin-responsiveness
	MBSI	1	1	Drought-responsiveness
	ERE	3	1	Ethylene-responsiveness
SlGH9-12	MYB	4	15	Abiotic stress responsiveness
	STRE	10		
	TC-rich repeats	1		
	LTR	1	1	Low-temperature responsiveness
	TGACG-motif	1	2	MeJA-responsiveness
	CGTCA-motif	1		
	F-box	2	1	Development and growth-related
	W box	1	1	Wound-responsiveness
	TCA	1	2	Salicylic acid-responsiveness

	TCA-element	1		
	AuxRR-core	1		
	TGA-element	1	2	Auxin-responsiveness
	WUN-motif	1	1	Wound-responsiveness
	ABRE4	2		
	ABRE3a	2	6	Absicid acid responsiveness
	ABRE	2		
	ERE	2	2	Ethylene-responsiveness
SlGH9-13	ABRE	2	2	Absicid acid responsiveness
	ERE	6	6	Ethylene-responsiveness
	MYB	1		
	MYC	2	5	Abiotic stress responsiveness
	STRE	2		
	W box	1	1	Wound-responsiveness
	O2-site	1	1	Development and growth-related
	TGA-element	1	1	Auxin-responsiveness
	TCA-element	2	2	Salicylic acid-responsiveness
SlGH9-14	P-box	2	2	Gibberellin-responsiveness
	W box	1	1	Wound-responsiveness
	ABRE	1	1	Absicid acid responsiveness
	MYC	6		
	STRE	1	8	Abiotic stress responsiveness
	MYB	1		
	TCA-element	2	2	Salicylic acid-responsiveness
	WRE3	1	1	Biotic stress responsiveness
	ERE	1	1	Ethylene-responsiveness
SlGH9-15	ERE	3	3	Ethylene-responsiveness
	TCA-element	2	2	Salicylic acid-responsiveness
	ABRE4	3		
	ABRE	12	18	Absicid acid responsiveness
	ABRE3a	3		
	MYC	6	12	Abiotic stress responsiveness
	MYB	6		
	W box	1	2	Wound-responsiveness
	WUN-motif	1		
	TGACG-motif	3	6	MeJA-responsiveness

	CGTCA-motif	3		
	WRE3	1	1	Biotic stress responsiveness
	MBS	3	4	Drought-responsiveness
	MBSI	1		
	AuxRR-core	1	1	Auxin-responsiveness
SlGH9-16	GARE-motif	1	1	Gibberellin-responsiveness
	MYC	5	19	Abiotic stress responsiveness
	STRE	6		
	MYB	8		
	ERE	1	1	Ethylene-responsiveness
	CAT-box	2	3	Development and growth-related
	O2-site	1		
	W box	1	1	Wound-responsiveness
	TCA	1	1	Salicylic acid-responsiveness
	CGTCA-motif	1	2	MeJA-responsiveness
	TGACG-motif	1		
	WRE3	1	1	Biotic stress responsiveness
SlGH9-17	MYB	5	12	Abiotic stress responsiveness
	MYC	7		
	ERE	5	5	Ethylene-responsiveness
	ABRE	1	3	Absicid acid responsiveness
	ABRE3a	1		
	ABRE4	1		
	LTR	1	1	Low-temperature responsiveness
	WUN-motif	1	1	Wound-responsiveness
	TGACG-motif	1	2	MeJA-responsiveness
	CGTCA-motif	1		
	TCA	1	1	Salicylic acid-responsiveness
	P-box	2	2	Gibberellin-responsiveness
SlGH9-18	TC-rich repeats	1	4	Abiotic stress responsiveness
	MYC	1		
	MYB	2		
	TGA-box	1	1	Auxin-responsiveness
	CCGTCC motif	3	8	MeJA-responsiveness
	CGTCA-motif	2		

	CCGTCC-box	3		
	TCA-element	1	1	Salicylic acid-responsiveness
	ERE	2	2	Ethylene-responsiveness
	MBS	1	1	Drought-responsiveness
	WUN-motif	1	1	Wound-responsiveness
	ABRE	1	1	Absicid acid responsiveness
SlGH9-19	MYC	5	8	Abiotic stress responsiveness
	MYB	3		
	GARE-motif	1	1	Gibberellin-responsiveness
	CAT-box	1	2	Development and growth-related
	F-box	1		
	ABRE3a	1	4	Absicid acid responsiveness
	ABRE	2		
	ABRE4	1		
	ERE	2	2	Ethylene-responsiveness
	WRE3	1	1	Biotic stress responsiveness

Table S6. Number of *cis*-elements in the promoters of *SlGH9* genes per category and class

	Class A						Class B										Class C		
***cis*-elements category**	*SlGH9-1*	SlGH9-4	SlGH9-6	SlGH9-7	SlGH9-8	SlGH9-17	SlGH9-2	SlGH9-5	SlGH9-9	SlGH9-10	SlGH9-11	SlGH9-12	SlGH9-14	SlGH9-15	SlGH9-16	SlGH9-18	SlGH9-3	SlGH9-13	SlGH9-19
Wound	0	4	3	2	1	1	0	3	1	0	0	2	1	2	1	1	2	1	0
Abiotic stress	10	12	15	11	11	12	13	8	8	5	11	15	8	12	19	4	11	5	8
MeJA	2	2	6	2	0	2	2	4	0	4	0	2	0	6	2	8	2	0	0
ABA	0	0	3	6	3	3	1	1	3	2	8	6	1	18	0	1	11	2	4
Giberellin	1	1	0	1	1	2	0	1	0	2	0	0	2	0	1	0	0	0	1
Drought	0	1	2	0	0	0	0	1	0	0	1	0	0	4	0	1	0	0	0
Devel-growth	0	0	1	0	2	0	1	2	3	0	0	1	0	0	3	0	0	1	2
Ethylene	2	0	3	2	0	5	6	4	1	0	0	2	1	3	1	2	0	6	2
SA	3	1	1	1	1	1	3	0	2	3	1	2	2	2	1	1	2	2	0
Biotic stress	0	0	0	0	0	0	0	0	0	0	1	0	1	1	1	0	0	0	1
Aux	0	1	0	0	0	0	0	0	0	0	1	2	0	1	0	1	0	1	0
Low temp	0	2	1	0	0	1	0	0	0	0	0	1	0	0	0	0	0	0	0

Table S7. Protein-protein interaction network (PPI) of the 10 selected SlGH9 proteins (SlGH9-1, SlGH9-2, SlGH9-4, SlGH9-7, SlGH9-11, SlGH9-12, SlGH9-13, SlGH9-15, SlGH9-17 and SlGH9-19) with interacting partner derived from the STRING database.

Protein	Interacting partner	Function	Score	PPI enrichment p-value
SlGH9-1	A0A3Q7EN49	Uncharacterized protein.	0.663	< 1.0e-16
	A0A3Q7GOT6	Uncharacterized protein.	0.633	
	A0A3Q7H063	Uncharacterized protein.	0.633	
	A0A3Q7H1P7	Uncharacterized protein; Belongs to the glycosyl hydrolase 3 family	0.633	
	A0A3Q7H2W5	Uncharacterized protein.	0.633	
	A0A3Q7HVR3	Uncharacterized protein.	0.633	
	A0A3Q7J0Z8	Uncharacterized protein.	0.633	
	A0A3Q7I5S7	Uncharacterized protein.	0.651	
	A0A3Q7EAX7	Uncharacterized protein; Belongs to the glycosyl hydrolase 1 family	0.650	
	A0A3Q7FS27	Uncharacterized protein.	0.650	
SlGH9-2	A0A3Q7ELP9	Protein kinase domain-containing protein.	0.803	0.00857
	A0A3Q7EN49	Uncharacterized protein.	0.663	
	A0A3Q7ERR2	BHLH domain-containing protein.	0.803	
	A0A3Q7EW07	Beta-galactosidase; Belongs to the glycosyl hydrolase 35 family.	0.664	
	A0A3Q7FCK4	BHLH domain-containing protein.	0.783	
	A0A3Q7GYH2	BHLH domain-containing protein.	0.72	
	A0A3Q7H324	CULLIN_2 domain-containing protein; Belongs to the cullin family.	0.668	
	A0A3Q7HRT8	BHLH domain-containing protein.	0.783	
	A0A3Q7HXB1	CULLIN_2 domain-containing protein; Belongs to the cullin family.	0.668	
	A0A3Q7IHK7	HECT domain-containing protein.	0.664	
SlGH9-4	A0A3Q7EN49	Uncharacterized protein.	0.663	< 1.0e-16
	A0A3Q7EN49	Uncharacterized protein.	0.663	

	A0A3Q7H063	Uncharacterized protein.	0.663	
	A0A3Q7H1P7	Uncharacterized protein; Belongs to the glycosyl hydrolase 3 family.	0.663	
	A0A3Q7H2W5	Uncharacterized protein.	0.663	
	A0A3Q7HVR3	Uncharacterized protein.	0.663	
	A0A3Q7J0Z8	Uncharacterized protein.	0.663	
	A0A3Q7EAX7	Uncharacterized protein; Belongs to the glycosyl hydrolase 1 family.	0.650	
	A0A3Q7FS27	Uncharacterized protein.	0.650	
	A0A3Q7HHG6	Uncharacterized protein; Belongs to the glycosyl hydrolase 1 family.	0.650	
SlGH9-7	A0A3Q7EN49	Uncharacterized protein.	0.663	< 1.0e-16
	A0A3Q7GOT6	Uncharacterized protein.	0.633	
	A0A3Q7H063	Uncharacterized protein.	0.633	
	A0A3Q7H1P7	Uncharacterized protein; Belongs to the glycosyl hydrolase 3 family	0.633	
	A0A3Q7H2W5	Uncharacterized protein.	0.633	
	A0A3Q7HVR3	Uncharacterized protein.	0.633	
	A0A3Q7J0Z8	Uncharacterized protein.	0.633	
	A0A3Q7I5S7	Uncharacterized protein.	0.651	
	A0A3Q7EAX7	Uncharacterized protein; Belongs to the glycosyl hydrolase 1 family	0.650	
	A0A3Q7FS27	Uncharacterized protein.	0.650	
SlGH9-11	A0A3Q7EFU1	Dihydrolipoamide acetyltransferase component of pyruvate dehydrogenase complex.	0.443	0.701
	A0A3Q7EN42	WD_REPEATS_REGION domain-containing protein.	0.495	
	A0A3Q7G7J2	Uncharacterized protein.	0.487	
	A0A3Q7HUB5	Uncharacterized protein; Belongs to the WD repeat SEC13 family.	0.409	
	A0A3Q7INM7	Protein kinase domain-containing protein; Belongs to the protein kinase superfamily.	0.489	
	A0A3Q7IY37	B30.2/SPRY domain-containing protein.	0.492	
	A0A3Q7JN10	Uncharacterized protein.	0.561	
SlGH9-12	LeEXP1	Expansin.	0.566	0.354
	A0A3Q7JN10	Uncharacterized protein.	0.561	
	XYL1	Fn3_like domain-containing protein.	0.544	

	SlIAA9	Auxin-responsive protein.	0.541	
	XTH5	GH16 domain-containing protein.	0.528	
	teg1A	Beta-galactosidase.	0.528	
	A0A3Q7EN42	WD_REPEATS_REGION domain-containing protein.	0.495	
	A0A3Q7IY37	B30.2/SPRY domain-containing protein.	0.492	
	A0A3Q7IIH1	Cellulase domain-containing protein.	0.490	
	A0A3Q7INM7	Protein kinase domain-containing protein.	0.489	
SlGH9-13	TBG6	Beta-galactosidase.	0.708	1.11e-16
	A0A3Q7EN49	Uncharacterized protein.	0.663	
	A0A3Q7GOT6	Uncharacterized protein.	0.663	
	A0A3Q7H063	Uncharacterized protein.	0.663	
	A0A3Q7H1P7	Uncharacterized protein; Belongs to the glycosyl hydrolase 3 family	0.663	
	A0A3Q7H2W5	Uncharacterized protein.	0.663	
	A0A3Q7HVR3	Uncharacterized protein.	0.663	
	A0A3Q7J0Z8	Uncharacterized protein.	0.663	
	A0A3Q7EAX7	Uncharacterized protein; Belongs to the glycosyl hydrolase 1 family	0.659	
	A0A3Q7FS27	Uncharacterized protein.	0.650	
SlGH9-15	A0A3Q7EAX7	Uncharacterized protein; Belongs to the glycosyl hydrolase 1 family.	0.655	< 1E-16
	A0A3Q7EN49	Uncharacterized protein.	0.663	
	A0A3Q7FS27	Uncharacterized protein.	0.655	
	A0A3Q7G0T6	Uncharacterized protein.	0.663	
	A0A3Q7H063	Uncharacterized protein.	0.663	
	A0A3Q7H1P7	Uncharacterized protein; Belongs to the glycosyl hydrolase 3 family.	0.663	
	A0A3Q7H2W5	Uncharacterized protein.	0.663	
	A0A3Q7HVR3	Uncharacterized protein.	0.663	
	A0A3Q7J0Z8	Uncharacterized protein.	0.663	
	A0A3Q7J8Z2	Uncharacterized protein; Belongs to the glycosyl hydrolase 1 family.	0.655	
SlGH9-17	A0A3Q7IIS9	Non-specific lipid-transfer protein; Plant non-specific lipid-transfer proteins transfer phospholipids as galactolipids across membranes.	0.716	8.18e-06

	A0A3Q7JDW8	May play a role in wax or cutin deposition in the cell walls of expanding epidermal cells and certain secretory tissues. Non-specific lipid-transfer protein; Plant non-specific lipid-transfer proteins transfer phospholipids as galactolipids across membranes. May play a role in wax or cutin deposition in the cell walls of expanding epidermal cells and certain secretory tissues.	0.716	
	A0A3Q7G6N9	Chitin-binding type-1 domain-containing protein.	0.715	
	A0A3Q7F6Q4	Uncharacterized protein.	0.714	
	A0A3Q7F6T3	Uncharacterized protein.	0.714	
	A0A3Q7FES4	Uncharacterized protein.	0.714	
	A0A3Q7I2L3	Uncharacterized protein.	0.714	
	A0A3Q7EAX7	Uncharacterized protein; Belongs to the glycosyl hydrolase 1 family.	0.650	
	A0A3Q7EN49	Uncharacterized protein.	0.650	
	A0A3Q7FS27	Uncharacterized protein.	0.650	
SlGH9-19	A0A3Q7EAX7	Uncharacterized protein; Belongs to the glycosyl hydrolase 1 family.	0.659	1.33E-11
	A0A3Q7EN49	Uncharacterized protein.	0.663	
	A0A3Q7ENQ6	F-box domain-containing protein.	0.729	
	A0A3Q7G0T6	Uncharacterized protein.	0.663	
	A0A3Q7H063	Uncharacterized protein.	0.663	
	A0A3Q7H1P7	Uncharacterized protein; Belongs to the glycosyl hydrolase 3 family.	0.663	
	A0A3Q7H2W5	Uncharacterized protein.	0.663	
	A0A3Q7HVR3	Uncharacterized protein.	0.663	
	A0A3Q7J0Z8	Uncharacterized protein.	0.711	
	A0A3Q7JG98	Uncharacterized protein; Belongs to the MIP/aquaporin (TC 1.A.8) family	0.711	
